# Amino acid sequence homology between thyroid autoantigens and central nervous system proteins: Implications for the steroid-responsive encephalopathy associated with autoimmune thyroiditis

**DOI:** 10.1016/j.jcte.2021.100274

**Published:** 2021-11-06

**Authors:** Salvatore Benvenga, Alessandro Antonelli, Poupak Fallahi, Carmen Bonanno, Carmelo Rodolico, Fabrizio Guarneri

**Affiliations:** a Endocrinology, Department of Clinical and Experimental Medicine, University of Messina, Via Consolare Valeria 1, 98125 Messina, Italy; b Interdepartmental Program on Molecular and Clinical Endocrinology and Women’s Endocrine Health, AOU Policlinico “G. Martino”, Via Consolare Valeria 1, 98125 Messina, Italy; c Master Program on Childhood, Adolescent and Women’s Endocrine Health, Via Consolare Valeria 1, 98125 Messina, Italy; d Department of Clinical and Experimental Medicine, University of Pisa, Via Paolo Savi 10, 56126 Pisa, Italy; e Neurology, Department of Clinical and Experimental Medicine, University of Messina, Via Consolare Valeria 1, 98125 Messina, Italy; f Dermatology, Department of Clinical and Experimental Medicine, University of Messina, Via Consolare Valeria 1, 98125 Messina, Italy

**Keywords:** AChR, acetylcholine receptors, AD, Alzheimer disease, AKRIAI, aldehyde reductase-I, ALS, amyotrophic lateral sclerosis, AT, autoimmune thyroiditis, BBB, blood-brain barrier, BLAST, Basic Local Alignment Search Tool, CCP, complement control protein, DDAHI, dimethylargininase-I, EGF, epidermal growth factor, GD, Graves' disease, GPCR, G protein-coupled receptors, HE, Hashimoto’s encephalopathy, HT, Hashimoto’s thyroiditis, LRR, leucine-rich repeats, MG, myasthenia gravis, MuSK, muscular tyrosin kinase receptors, NMJ, neuromuscular junction, SREAT, steroid-responsive encephalopathy associated with autoimmune thyroiditis, TAb, anti-thyroid antibodies, Graves’ disease, Hashimoto’s encephalopathy, Thyroglobulin, Thyroperoxidase, Thyrotropin receptors, Bioinformatics

## Abstract

A few patients with Hashimoto’s thyroiditis or Graves’ disease develop a multiform syndrome of the central nervous system (CNS) termed Hashimoto’s encephalopathy or steroid-responsive encephalopathy associated with autoimmune thyroid disease (HE/SREAT). They have high levels of thyroid autoantibodies (TgAb, TPOAb and/or TSH-R-Ab) in blood and cerebrospinal fluid. Autoantibodies against alpha-enolase, aldehyde reductase-I (AKRIA) and/or dimethylargininase-I (DDAHI), proteins expressed in the CNS among other tissues, were detected in the blood and, when searched, in the cerebrospinal fluid of HE/SREAT patients. Recently, we reported that alpha-enolase, AKRIA and DDAHI share local sequence homology with each of the three autoantigens (TgAb, TPOAb, TSH-R-Ab), often in epitope-containing segments of the thyroid autoantigens. We hypothesized that there might be additional CNS-expressed proteins homologous to thyroid autoantigens, possibly overlapping known epitopes of the thyroid autoantigens. We used bioinformatic methods to address this hypothesis.

Six, 27 and 47 of 46,809 CNS-expressed proteins share homology with TSH-R, Tg and TPO, respectively. The homologous regions often contain epitopes, and some match regions of thyroid autoantigens which have homology with alpha-enolase, AKRIA and/or DDAHI. Several of the aforementioned proteins are present in CNS areas that show abnormalities at neuroimaging in HE/SREAT patients. Furthermore, autoantibodies against some of the said six, 27 and 47 proteins were reported to be associated with a number of autoimmune diseases.

Not only we validated our hypothesis, but we think that such a variety of potential CNS targets for thyroid Ab against epitopes contained in regions that have local homology with CNS proteins may explain the polymorphic phenotypes of HE/SREAT. Only when elevated amounts of these Ab are synthesized and trespass the blood-brain barrier, HE/SREAT appears. This might explain why HE/SREAT is so relatively rare.

## Highlights

• Alpha-enolase, aldehyde reductase-I and dimethylargininase-I are SREAT autoantigens.
• Molecular mimicry between thyroid and CNS autoantigens is hypothesized in SREAT.
• Homology with TSH-R, Tg and TPO exists for 6, 27 and 47 of 46,809 CNS-proteins.
• The above homologies are often in epitope-containing parts of thyroid autoantigens.
• Most of the above proteins are expressed in CNS regions which are altered in SREAT.

## Introduction

Hashimoto’s encephalopathy (HE) was initially described in 1966 in association with Hashimoto’s thyroiditis (HT) [1], and later found to be associated, although less frequently, with the other autoimmune thyroiditis (AT): Graves' disease (GD). Because, regardless of the HT or GD association, encephalopathy is very sensitive to corticosteroid therapy, another denomination is steroid-responsive encephalopathy associated with autoimmune thyroiditis (SREAT). SREAT represents a rare complication of autoimmune thyroiditis [2] and may precede it even by years, similar to thyroid eye disease in patients with Graves disease [3], [4]. SREAT patients have abnormal electroencephalography and increased concentration of proteins/immunoglobulins G (IgG) in the cerebrospinal fluid, which can be observed in 90% and 80% of patients, respectively, but these findings are not specific of the disease [5]. Serum anti-thyroid antibodies (TAb) are typically elevated in SREAT patients, but their levels do not correlate with either severity or any specific clinical presentation.

Between 2002 and 2008, three autoantigens shared by the central nervous system (CNS) and the thyroid, and targeted by autoantibodies specifically present in SREAT patients, were identified: alpha-enolase, dimethylargininase-I (DDAHI) and aldehyde reductase-I (AKRIAI) [6], [7], [8]. This discovery led to the idea that autoimmunity against autoantigens common to CNS and thyroid could be one of the pathogenetic mechanisms of SREAT, in addition to the action of antithyroid autoantibodies on Tg, TPO and TSH-R expressed in the central nervous system [9].

In 2003, a paper described one patient with HE and reviewed the HE literature (85 patients who met their inclusion criteria out of “*105 patients with brain dysfunction associated with possible Hashimoto thyroiditis*”) [10]. This paper reported that pathologic findings were available for only three HE patients (one based on necropsy and two based on brain biopsy) [10]. In one patient, autopsy revealed lymphocytic infiltration in the brainstem (including its veins and venules), leptomeninx of the cortex, and cerebellum [11]. In the other two patients, biopsy revealed lymphocytic infiltration of the walls of many small arterioles and venules [12], and perivascular cuffs of lymphocytic cells [10]. Quite interestingly, Chong et al. [10] wrote that it could not be excluded that the high serum levels of TAb found in HE patients were originated by reaction to proteins (viral, bacterial, or toxic) causing brain damage or brain antigens released after injury, but there were no known proteins in the above categories with structural similarity to thyroid autoantigens.

For sake of completeness, we should note that Chong et al. [10] missed three patients. One was a French patient [13], in whom postmortem neuropathology demonstrated nonspecifically activated microglia. The second was a Japanese patient [14], in whom autopsy revealed no evidence of CNS vasculitis or other brain abnormalities. The third was an American patient with a questionable 7-mm area of the left medial frontal cortex at MRI [15]. Biopsy revealed moderate gliosis, some perivascular lymphoid cells and macrophages, scattered microglia in the parenchyma, but not vasculitis or microglial nodules [15].

In subsequent years, postmortem examination in HE patients demonstrated “*mild perivascular lymphocytic infiltration throughout the brain and leptomeninges plus diffuse gliosis of gray matter in the cortex, basal ganglia, thalami, hippocampi, and, to a lesser extent, the parenchymal white matter*” [16]. Biopsy of other HE patients revealed: [i] “*patchy myelin pallor, scant perivascular chronic inflammation, mild gliosis, and microglial activation*” [17]; [ii] primary vasculitis of the CNS [18]; [iii]“*diffuse gliosis and perivascular lymphocyte infiltration with CD3 + T-cell predominance, … with no signs of a brain tumor*” in a patient with a tumor-like lesion of the left caudate nucleus, “*suggesting cerebral vasculitis as an underlying etiology*” [19]; [iv] non-vasculitic autoimmune inflammatory meningoencephalitis [20]; [v] reactive gliosis, angiogenesis, swollen vascular endothelial cells, mild lymphocyte infiltration (almost exclusively T cells) around small vessels [21].

Molecular mimicry between thyroid autoantigens and other autoantigens was mentioned by several authors as a possible clinically relevant causal mechanism of extrathyroid manifestations of thyroid autoimmunity, including some neurological and pychiatric disorders [22], [23], [24].

Just very recently, we demonstrated that there is striking local homology between thyroid autoantigens and the three HE/SREAT-autoantigens [25]. Particularly, Tg was homologous to 10 regions of alpha-enolase, 8 regions of AKRIAI, and 5 regions of DDAHI. TPO was homologous to 6 regions of alpha-enolase, 7 regions of AKRIAI, and 3 regions of DDAHI. Finally, TSH-R was homologous to 4 regions of alpha-enolase, 5 regions of AKRIAI, and 2 regions of DDAHI. Importantly, in regard to alpha-enolase (the sole of the three HE/SREAT autoantigens for which epitopes have been characterized), a total of 5 regions homologous to Tg, one region homologous to TPO, and one region homologous to TSH-R fell within, or adjacent to, epitopes of the protein. From the opposite perspective, a total of 4 regions of Tg, 5 of TPO and 2 of TSH-R homologous to alpha-enolase contained epitopes. Epitopes in each of the three thyroid autoantigens were present also in their regions that were homologous to regions of AKRIAI and DDAHI [25]. In brief, we provided some indirect evidence that a number of regions of homologies were relevant for the autoimmunity associated with HE/SREAT.

We hypothesized that alpha-enolase, AKRIAI and DDAHI might be the classic “tip of the iceberg”, *viz*. we hypothesized that there could be more proteins expressed in the CNS, not necessarily in a CNS-restricted expression mode, which share homology with at least one of the three thyroid autoantigens. Applying the same bioinformatic approach used for alpha-enolase, AKRIAI and DDAHI, we searched for such homologies.

## Material and methods

We used our standard procedure, as in previous bioinformatics papers [25], [26], [27], [28], [29], [30], [31]. We retrieved the amino acid sequence of the precursors of the three “classical” human thyroid autoantigens, i.e. TSH-R (accession number P16473), Tg (accession number NP_003226) and TPO (accession number AAA61217) from the Entrez Protein database (https://www.ncbi.nlm.nih.gov/protein). Next, we probed each of these three autoantigens for amino acid sequence homology with human proteins of the same database whose records contained the term “brain” or “central nervous system”. Proteins labeled as “incomplete” or “hypothetical” were excluded. We also excluded alpha-enolase, AKRIAI and DDAHI, since they were investigated in our previous paper [25]. The Protein BLAST (Basic Local Alignment Search Tool) software version 2.8.0+ [32] was used to perform the comparison. Analysis was made with the standard parameters of the program, and only results with E < 10 were considered. Finally, the records of the proteins identified were manually reviewed, to exclude those not expressed in the CNS (the presence of the terms “brain” and/or “central nervous system” in the record was sometimes incidental, not related to the actual localization of the protein).

As also done previously [25], [26], [27], [28], [29], [30], [31], we verified the immunological relevance of the homologies selected, checking for their possible overlap(s) with known epitopes of TSH-R, Tg and TPO [26], [27], [28], [29], [30], [31], [33], [34], [35], [36]. To strengthen the immunological relevance of the homologies that we found, we searched the literature for the presence of serum autoantibodies against each of the thyroid autoantigen-homologous proteins in autoimmune diseases, including thyroid autoimmune diseases. To this aim, we searched in the PubMed database using the search string “(autoanti* OR autoimm* OR autoreact*) AND” followed by the name of each protein, and manually revised the results to select only relevant original articles.

To quickly know (i) which areas of the CNS express each of the proteins that we found to be thyroid autoantigen-homologous, and (ii) whether the thyroid gland also expressed these proteins, we probed the Expression Atlas (https://www.ebi.ac.uk/gxa/home) [37].

## Results

### CNS-expressed proteins found to be homologous to thyroid autoantigens

Table 1, Table 2 and Table 3 list which of the 46,809 CNS-expressed proteins in our databank, were homologous to TSH-R, Tg and TPO, respectively. There were 46 proteins (∼0.1%), 27 (∼0.06%) and 47 proteins (∼0.1%) that shared homology with TSH-R, Tg and TPO, respectively. Table 1, Table 2, Table 3 illustrate the span of the homologous segments, the degree of amino acid identity and overall amino acid homology (namely, identity plus similarity).

**Table 1:** Homologies between TSH-R and proteins from brain or central nervous system.

	**Protein [Entrez Protein GI accession number]**	**Protein segment**	**TSH-R segment**	**Identity**	Overall homology*	**E value**	Coincidences with**		
1	Leucine-rich repeat-containing G-protein coupled receptor 4 (LGR4) [157694513]	20–253	20–252	24%	39%	1.19 × 10^−4^	**Eno**		***D***
		177–815	52–692	24%	44%	6.95 × 10^−45^	**Eno**	*A*	***D***
2	Leucine-rich repeat-containing G-protein coupled receptor 5 (LGR5) [4504379]	234–868	32–732	24%	42%	1.25 × 10^−43^	**Eno**	*A*	***D***
3	Relaxin receptor 2/Leucine-rich repeat-containing G-protein coupled receptor 8 (LGR8) [18677729]	115–708	29–695	22%	40%	7.82 × 10^−29^	**Eno**	*A*	***D***
4	Relaxin receptor 1/Leucine-rich repeat-containing G protein-coupled receptor 7 (LGR7) [359279868]	182–738	182–710	25%	44%	1.75 × 10^−34^	**Eno**	*A*	***D***
5	Chondroadherin [153251229]	24–219	28–250	21%	44%	0.013	**Eno**		***D***
6	Leucine-rich repeat and immunoglobulin-like domain-containing nogo receptor-interacting protein 2 precursor (LINGO2) [22749183]	26–205	22–254	25%	43%	0.028	**Eno**		***D***
7	Somatostatin receptor type 2 [4557859]	24–322	395–688	23%	41%	1.14 × 10^−9^	**Eno**	*A*	
8	Neuropeptide Y receptor type 1 [4505445]	50–331	424–689	24%	41%	2.07 × 10^−8^	**Eno**	*A*	
9	Apelin receptor [4885057]	38–318	424–687	25%	42%	2.78 × 10^−8^	**Eno**	*A*	
10	Neuromedin-K receptor/Neurokinin B receptor/Tachykinin receptor 3 [7669548]	84–382	418–696	26%	43%	4.26 × 10^−8^	**Eno**	*A*	
11	Free fatty acid receptor 3 [4885329]	88–334	494–731	20%	41%	5.11 × 10^−7^	**Eno**	*A*	
12	Melanopsin/Opsin-4 [15150803]	73–386	417–710	22%	36%	1.60 × 10^−6^	**Eno**	*A*	
13	G-protein coupled estrogen receptor 1/Membrane estrogen receptor [4504091]	68–332	423–686	23%	41%	5.02 × 10^−6^	**Eno**	*A*	
14	Alpha-1A adrenergic receptor [111118992]	9–354	393–706	20%	38%	1.71 × 10^−5^	**Eno**	*A*	
15	Vasopressin V1a receptor [4502331]	64–358	427–688	21%	39%	3.46 × 10^−5^	**Eno**	*A*	
16	Probable G-protein coupled receptor 34 [4885319]	26–367	370–727	19%	40%	3.86 × 10^−5^	**Eno**	*A*	
17	G-protein coupled receptor 26 [23592220]	79–200	494–609	26%	47%	7.64 × 10^−5^	**Eno**	*A*	
18	Orexin 2 receptor [1285033761]	71–163	424–523	28%	50%	8.46 × 10^−5^			
19	Oxytocin receptor [32307152]	56–357	431–711	23%	40%	1.28 × 10^−4^	**Eno**	*A*	
20	Orexin receptor type 1 [222080095]	63–169	431–546	28%	44%	4.14 × 10^−4^			
21	Galanin receptor type 2 [4503905]	37–302	426–688	22%	39%	6.07 × 10^−4^	**Eno**	*A*	
22	GPER protein [52350636]	68–274	423–639	24%	42%	6.90 × 10^−4^	**Eno**	*A*	
23	N/OFQ opioid receptor [385252102]	135–401	424–686	22%	41%	0.002	**Eno**	*A*	
24	Type 2 angiotensin II receptor [23238240]	103–350	481–707	24%	41%	0.002	**Eno**	*A*	
25	Alpha-1B adrenergic receptor [4501959]	57–357	426–687	19%	37%	0.006	**Eno**	*A*	
26	Mu opioid receptor [119568090]	142–453	424–744	20%	39%	0.011	**Eno**	*A*	
27	Melanin-concentrating hormone receptor 1 [397487122]	119–393	424–691	22%	40%	0.013	**Eno**	*A*	
28	Bombesin receptor subtype-3 [4502455]	60–339	427–687	19%	40%	0.023	**Eno**	*A*	
29	Neuropeptide Y receptor type 5 [5453796]	5–93	381–466	27%	50%	0.029		*A*	
30	C3a anaphylatoxin chemotactic receptor [4757888]	331–444	576–687	22%	44%	0.151		*A*	
31	Substance-P receptor/Tachykinin receptor 1 [4507343]	54–163	436–554	25%	41%	0.365			
32	Proteinase-activated receptor 2 [34577052]	77–352	416–686	21%	39%	0.394	**Eno**	*A*	
33	Trace amine-associated receptor 6 (TaR-6) [28173558]	35–135	417–524	30%	53%	0.463			
34	Urotensin-2 receptor/G-protein coupled receptor 14 [9506745]	115–323	487–686	23%	42%	0.515	**Eno**	*A*	
35	Nociceptin receptor [974065167]	132–322	508–686	25%	44%	0.779	**Eno**	*A*	
36	G-protein coupled receptor 24 [56554976]	149–267	584–691	24%	47%	0.812		*A*	
37	C–C chemokine receptor type 7/Epstein-Barr virus-induced G-protein coupled receptor 1/MIP-3 beta receptor [4502641]	320–374	672–725	29%	49%	0.941			
38	Olfactory receptor 2A14 [48717236]	96–164	494–562	30%	47%	1.001			
39	Vasopressin V2 receptor [4557345]	267–326	620–679	30%	45%	1.119		*A*	
40	Neuropeptide S receptor [46395496]	54–336	419–684	21%	42%	1.139	**Eno**	*A*	
41	Trace amine-associated receptor 8 (TaR-8) [16751917]	40–100	423–483	38%	60%	1.342			
42	Neuropeptides B/W receptor type 2 [30581164]	58–326	427–688	24%	41%	2.476	**Eno**	*A*	
43	Olfactory receptor 2 J3 [185134902]	39–156	426–550	24%	44%	2.929			
44	G protein-coupled receptor [953233]	299–318	670–689	55%	75%	7.670			
45	Oxoglutarate (alpha-ketoglutarate) receptor 1 [52426789]	18–313	402–689	23%	38%	7.822	**Eno**	*A*	
46	5-hydroxytryptamine receptor 7 (5-HT7) [10880129]	111–389	445–683	20%	36%	8.232	**Eno**	*A*	

*Identical plus similar amino acids.

**Coincidences with segments of TSH-R homologous to known autoantigens of Hashimoto’s encephalopathy, i.e. alpha-enolase (**Eno**), AKRIAI (*A*) and DDAHI (***D***). Segments 149–161 and 560–575 of TSH-R are homologous to segments 40–52 and 284–299 of alpha-enolase, respectively. Segments 360–415, 396–402, 555–563 and 620–676 of TSH-R are homologous to segments 89–141, 258–264, 14–22, and 268–325 of AKRIAI, respectively. Segments 141–148 and 263–292 of TSH-R are homologous to segments 242–249 and 258–283 of DDAHI, respectively [25].

**Table 2:** Homologies between thyroglobulin (Tg) and proteins from brain or central nervous system.

	Protein [Entrez Protein GI accession number]	Protein segment	Tgs egment	Identity	Overall homology*	E value	Coincidences with**		
1	Nidogen-1/Entactin [115298674]	847–919	660–726	35%	52%	1.23 × 10^−5^			
		849–917	96–161	39%	50%	1.61 × 10^−5^			
		859–922	1015–1076	43%	58%	8.51 × 10^−7^			
		867–925	1159–1216	45%	53%	4.60 × 10^−4^	**Eno**		
		874–919	315–358	45%	58%	0.002			
		880–919	882–921	35%	52%	0.281			
2	Testican-1/Protein SPOCK [4759164]	281–368	972–1062	29%	46%	6.03 × 10^−5^			
		312–385	298–367	37%	52%	2.42 × 10^−5^	**Eno**		
		313–372	96–155	35%	51%	0.001			
		333–372	616–653	50%	67%	7.80 × 10^−5^			
		333–376	48–92	40%	62%	0.001			
3	Testican-2/SPARC/osteonectin, CWCV, and Kazal-like domains proteoglycan 2 (SPOCK2) [7662036]	312–374	33–89	39%	52%	3.23 × 10^−4^		*A*	
		325–376	1019–1073	36%	54%	0.017			
		327–377	1160–1211	47%	64%	6.91 × 10^−5^	**Eno**		
		332–376	615–658	47%	63%	2.19 × 10^−4^			
		333–374	116–157	45%	59%	1.94 × 10^−4^			
		333–374	315–355	50%	66%	5.24 × 10^−5^			
4	SPARC-related modular calcium-binding protein 1/ Secreted modular calcium-binding protein 1 (SMOC-1) [11545873]	54–149	50–151	28%	40%	0.034			
		54–158	1106–1210	30%	39%	1.39 × 10^−4^	**Eno**	*A*	
		95–149	34–83	41%	54%	2.69 × 10^−4^		*A*	
		114–294	1027–1212	28%	42%	1.03 × 10^−9^	**Eno**	*A*	
		116–292	181–358	25%	39%	8.360	**Eno**		
		119–317	881–1110	26%	38%	1.73 × 10^−6^		*A*	
		227–272	34–73	30%	52%	6.927		*A*	
		227–340	96–212	26%	39%	0.221			
		239–295	609–661	40%	54%	0.031			
5	Testican-3 [3581970]	300–384	17–95	31%	43%	2.80 × 10^−4^	**Eno**		
		311–373	652–717	30%	52%	0.029			
		317–376	96–155	38%	46%	9.63 × 10^−4^			
		318–372	999–1062	42%	51%	1.42 × 10^−5^			
		337–376	315–353	45%	57%	0.018			
		337–376	616–653	42%	62%	0.005			
		337–376	1165–1205	48%	60%	5.46 × 10^−4^	**Eno**		
6	SPARC-related modular calcium-binding protein 2/ Secreted modular calcium-binding protein 2 (SMOC-2) [262050673]	91–154	598–659	34%	56%	1.05 × 10^−5^			
		105–153	311–358	36%	51%	0.043			
		109–156	116–163	39%	52%	0.002			
		109–251	48–194	25%	40%	0.017			
		109–301	1027–1230	28%	43%	4.21 × 10^−13^		*A*	
		198–327	78–210	26%	40%	0.030			
		233–295	613–672	33%	50%	0.012			
7	Insulin-like growth factor-binding protein 5 [10834982]	210–265	611–660	33%	51%	0.033			
		215–253	315–348	41%	56%	0.581			
8	Signal peptide, CUB and EGF-like domain-containing protein 1 [120587029]	636–752	1427–1532	29%	36%	0.106			
9	Ephrin type-B receptor 2 [822606583]	269–312	1473–1531	35%	44%	2.342			
10	Ephrin type-B receptor 6 [294862532]	311–335	1470–1494	48%	60%	6.469			
11	Ephrin type-A receptor 7 [568599847]	260–319	1457–1531	28%	42%	7.379			
12	Acetylcholinesterase (Yt blood group) [219518823]	46–573	2211–2728	32%	50%	4.79 × 10^−66^		*A*	***D***
13	Butyrylcholinesterase [1073548962]	9–527	2204–2722	29%	47%	2.43 × 10^−61^		*A*	***D***
14	Neuroligin-3 [262359974]	66–596	2225–2730	30%	46%	1.36 × 10^−52^		*A*	***D***
15	Neuroligin-4, X-linked [24308209]	70–539	2225–2671	29%	48%	2.60 × 10^−51^		*A*	***D***
16	Neuroligin-4, Y-linked [256222771]	70–539	2225–2671	29%	48%	2.98 × 10^−51^		*A*	***D***
17	Neuroligin-1 [1478051093]	77–546	2225–2671	31%	48%	1.49 × 10^−49^		*A*	***D***
18	Carboxylesterase 3 (CES3) [297747275]	38–550	2204–2724	31%	44%	3.73 × 10^−49^		*A*	***D***
19	Cocaine esterase [1463570077]	35–526	2204–2722	29%	45%	2.57 × 10^−47^		*A*	***D***
20	Carboxylesterase 5A [298231153]	83–580	2225–2730	28%	44%	1.93 × 10^−42^		*A*	***D***
21	Neuroligin-2 [30840978]	66–550	2225–2671	28%	43%	7.69 × 10^−42^		*A*	***D***
22	Brain carboxylesterase hBr3 [6009628]	21–549	2197–2720	28%	44%	2.70 × 10^−40^		*A*	***D***
23	Liver carboxylesterase 1/Acyl-coenzyme A:cholesterol acyltransferase/Brain carboxylesterase hBr1/Cocaine carboxylesterase/Egasyn/Methylumbelliferyl-acetate deacetylase 1/Monocyte/macrophage serine esterase/Retinyl ester hydrolase/Serine esterase 1/Triacylglycerol hydrolase [68508965]	21–552	2197–2723	28%	43%	9.33 × 10^−40^		*A*	***D***
24	KIAA1480 protein, partial [7959221]	36–470	2298–2730	28%	46%	8.41 × 10^−39^		*A*	***D***
25	Carboxylesterase 4A [1419235141]	30–509	2203–2669	29%	44%	4.62 × 10^−37^		*A*	***D***
26	Carboxylesterase 8 (CES8) [40555853]	34–390	2318–2669	28%	44%	5.22 × 10^−24^		*A*	***D***
27	KIAA1366 protein, partial [7243113]	1–265	2409–2671	22%	39%	0.002		*A*	***D***

*Identical plus similar amino acids.

**Coincidences with segments of Tg homologous to known autoantigens of Hashimoto’s encephalopathy, i.e. alpha-enolase (**Eno**), AKRIAI (*A*) and DDAHI (***D***). Segments 298–329, 1171–1186, 1315–1337 and 1368–1385 of Tg are homologous to segments 18–48, 208–223, 375–395 and 280–297 of alpha-enolase, respectively. Segments 31–90, 1086–1114, 1107–1129 and 2612–2668 of Tg are homologous to segments 178–227, 111–140, 6–26 and 86–124 of AKRIAI, respectively. Segments 1597–1612, 2277–2286 and 2605–2617 of Tg are homologous to segments 64–81, 218–227 and 230–242 of DDAHI, respectively [25].

**Table 3:** Homologies between thyroid peroxidase (TPO) and proteins from brain or central nervous system.

	Protein [Entrez Protein GI accession number]	Protein segment	TPO segment	Identity	Overall homology*	E value	Coincidences with**		
1	Peroxidasin homolog/Melanoma-associated antigen MG50/Vascular peroxidase 1 [109150416]	604–1314	8–734	41%	58%	1.89 × 10^−175^	**Eno**	*A*	***D***
2	Peroxidasin-like protein [633365073]	516–1201	40–734	38%	55%	5.01 × 10^−150^	**Eno**	*A*	***D***
3	Prostaglandin G/H synthase 2/Cyclooxygenase-2 [4506265]	208–340	318–459	28%	43%	1.50 × 10^−8^		*A*	
4	Prostaglandin G/H synthase 1/Cyclooxygenase-1 [18104967]	227–518	324–650	22%	40%	1.35 × 10^−5^	**Eno**	*A*	***D***
5	Fibrillin-1/Asprosin/Epididymis secretory sperm binding protein [311033452]	515–572	768–840	32%	47%	0.011			
		570–613	794–840	36%	55%	0.129			
		611–655	794–841	43%	56%	3.33 × 10^−4^			
		723–765	796–840	37%	55%	0.015			
		908–952	794–840	36%	51%	0.063			
		1024–1070	792–840	38%	48%	0.005			
		1068–1104	794–832	38%	51%	1.620			
		1174–1238	774–840	29%	46%	1.196			
		1216–1280	775–840	33%	52%	9.48 × 10^−4^			
		1344–1404	776–840	38%	55%	4.31 × 10^−5^			
		1392–1455	784–847	27%	50%	0.635			
		1645–1692	793–843	35%	45%	0.166			
		1888–1930	793–840	37%	50%	2.828			
		1928–1973	794–840	53%	63%	8.86 × 10^−7^			
		1973–2055	752–840	31%	47%	2.29 × 10^−5^			
		2129–2199	740–832	30%	45%	0.005			
		2244–2291	793–840	37%	52%	2.02 × 10^−4^			
		2289–2334	794–841	33%	47%	0.546			
		2413–2507	768–862	34%	46%	3.61 × 10^−5^			
		2522–2567	794–840	36%	46%	0.340			
		2575–2648	766–840	30%	47%	5.324			
		2646–2678	794–829	47%	58%	0.198			
6	Adhesion G protein-coupled receptor E2/EGF-like module receptor 2/CD312 [23397681]	65–101	794–830	40%	59%	0.030			
		158–191	791–825	54%	62%	1.82 × 10^−4^			
		209–240	793–825	45%	63%	0.155			
7	Protocadherin Fat 4 [165932370]	3799–3897	746–838	34%	41%	3.33 × 10^−4^			
8	Low-density lipoprotein receptor-related protein 4 (LRP-4) [157384998]	359–433	747–838	33%	46%	4.08 × 10^−4^			
9	Latent-transforming growth factor beta-binding protein 4 (LTBP4) [110347431]	355–397	794–839	36%	54%	0.431			
		585–636	794–847	38%	50%	0.506			
		627–671	794–840	42%	51%	1.506			
		750–794	794–840	44%	59%	0.064			
		872–920	790–840	43%	50%	0.023			
		1047–1091	794–840	51%	61%	5.63 × 10^−4^			
		1539–1604	764–825	37%	55%	1.419			
10	Fibrillin-3 [56237021]	487–557	794–866	36%	46%	0.004			
		570–614	794–841	39%	54%	0.174			
		681–724	795–840	39%	56%	0.323			
		763–816	793–852	35%	46%	5.987			
		867–911	794–840	44%	53%	0.396			
		982–1028	792–840	42%	44%	0.874			
		1153–1196	794–840	38%	48%	1.322			
		1169–1238	770–840	35%	47%	0.074			
		1443–1487	794–841	41%	52%	0.992			
		1884–1930	794–841	43%	52%	0.041			
		1959–2012	786–841	35%	51%	0.217			
		2083–2148	795–862	35%	45%	0.196			
		2204–2236	793–825	50%	64%	1.605			
		2368–2468	762–862	31%	42%	0.001			
		2483–2528	794–840	41%	50%	5.275			
		2536–2601	766–831	39%	52%	0.078			
		2598–2640	786–829	47%	56%	4.494			
11	Latent-transforming growth factor beta-binding protein 1 (LTBP-1) [290457687]	902–979	785–862	28%	42%	0.034			
		1074–1286	626–840	24%	36%	0.013	**Eno**		
		1200–1244	794–840	43%	56%	0.001			
		1436–1507	768–839	28%	45%	0.440			
		1621–1706	738–839	29%	36%	2.230			
12	Seizure related 6-like protein 2 [608785583]	541–610	736–802	33%	45%	0.003			
13	CUB and sushi domain-containing protein 1 [259013213]	1200–1282	739–805	29%	38%	0.968			
		2478–2555	727–797	34%	48%	0.003			
14	C-type lectin domain family 14 member A/Epidermal growth factor receptor 5 (EGFR-5) [28269707]	256–290	808–842	51%	60%	0.004			
15	fibrillin 1 variant, partial [62087260]	438–490	786–840	38%	54%	0.005			
16	Multiple epidermal growth factor-like domains protein 6 [110347457]	247–324	745–838	34%	45%	0.008			
17	Seizure 6-like protein/KIAA0927 protein [296179442]	392–449	741–795	34%	48%	0.009			
18	Cadherin EGF LAG seven-pass G-type receptor 2/Cadherin family member 10/Flamingo homolog 3 [13325064]	1296–1351	807–862	40%	50%	0.011			
19	Low-density lipoprotein receptor-related protein 2 (LRP-2) [126012573]	1388–1428	794–838	40%	51%	0.386			
		3136–3191	767–838	31%	44%	0.550			
		4000–4054	789–845	38%	49%	0.011			
20	EGF-containing fibulin-like extracellular matrix protein 2 [14714634]	121–164	794–840	44%	48%	0.272			
		141–203	772–840	34%	50%	0.012			
		263–319	776–829	36%	50%	0.120			
21	Nephronectin/Preosteoblast EGF-like repeat protein with MAM domain/EGFL6-like [75709198]	212–259	794–847	38%	61%	0.016			
22	Complement component C1q receptor/CD93 [88758613]	326–369	766–825	38%	46%	5.378			
		383–427	794–840	45%	56%	0.016			
		410–468	767–839	34%	45%	0.367			
23	Fibulin 5 [19743803]	113–161	768–831	35%	48%	0.019			
24	Tolloid-like protein 1 [22547221]	567–614	789–838	42%	50%	0.020			
25	EGF-containing fibulin-like extracellular matrix protein 1 [86788015]	204–254	786–840	38%	52%	0.023			
26	Signal peptide, CUB and EGF-like domain-containing protein 1 [120587029]	64–117	786–840	29%	49%	1.882			
		270–323	786–840	42%	50%	0.023			
		360–407	794–844	33%	56%	3.772			
27	Latent-transforming growth factor beta-binding protein 1 (LTBP1) [219518146]	576–665	785–881	26%	40%	0.032			
28	KIAA1237 protein, partial [34327974]	912–944	796–831	47%	58%	0.040			
29	Vitamin K-dependent protein S [192447438]	137–201	776–840	32%	50%	0.041			
30	Protein HEG homolog 1 [153792110]	1025–1057	796–831	47%	58%	0.047			
31	Low-density lipoprotein receptor-related protein 1B (LRP-1B) [93102379]	96–155	793–840	28%	41%	5.824			
		104–193	745–838	28%	45%	0.049			
		2909–2968	793–840	31%	50%	0.817			
		2966–3002	794–834	46%	63%	0.081			
32	P-selectin (CD62P)/Granule membrane protein 140/Leukocyte-endothelial cell adhesion molecule 3/Platelet activation dependent granule-external membrane protein [215274139]	531–621	759–843	31%	47%	0.053			
33	Fibulin-1 (FIBL-1) [215274249]	475–552	752–824	31%	46%	0.065			
34	Fibulin 1 [18490682]	189–257	755–834	35%	45%	0.257			
		354–405	794–846	41%	47%	0.066			
		390–441	787–840	37%	51%	0.404			
35	Protein kinase C-binding protein NELL2 [223029476]	461–500	794–834	43%	58%	0.067			
36	NOTCH4 protein [187954607]	192–230	795–838	43%	50%	6.636			
		272–342	756–825	32%	46%	0.077			
37	complement receptor type 2 [54792123]	398–467	731–795	33%	43%	2.909			
		935–970	764–798	50%	58%	0.103			
38	dual oxidase 2 precursor variant, partial [62087600]	59–106	650–695	33%	60%	0.113			
39	Nidogen-1/Entactin [115298674]	800–840	794–839	43%	50%	0.132			
40	CSMD2 protein [62954774]	2404–2593	627–802	25%	38%	0.135	**Eno**		
41	Cysteine-rich with EGF-like Domains 2 (CRELD2) beta [67511376]	202–266	767–839	35%	45%	0.135			
42	Endosialin/CD248 [9966885]	283–356	755–844	26%	38%	0.203			
43	Epidermal growth factor-like protein 7 [7705889]	137–185	796–847	40%	50%	0.208			
44	Prolow-density lipoprotein receptor-related protein 1/ Alpha-2-macroglobulin receptor/Apolipoprotein E receptor/CD91 [126012562]	148–188	794–838	42%	53%	2.343			
		2941–3012	797–863	35%	43%	0.251			
45	CUB and sushi domain-containing protein 3 [38045888]	2874–2939	741–806	34%	44%	0.260			
46	Thrombospondin-3 [6005902]	367–397	793–823	41%	64%	0.268			
47	Epidermal growth factor-like protein 6 [13124888]	93–135	795–841	42%	53%	1.360			
		217–252	794–830	43%	56%	0.270			
48	Mutant p53 binding protein 1 variant, partial [62087822]	254–293	796–840	35%	53%	0.782			

*Identical plus similar amino acids.

**Coincidences with segments of TPO homologous to known autoantigens of Hashimoto’s encephalopathy, i.e. alpha-enolase (**Eno**), AKRIAI (*A*) and DDAHI (***D***). Segments 603–627, 609–623, 637–659, 700–722, 710–721 of TPO are homologous to segments 261–281, 227–241, 211–233, 243–265, 346–357 of alpha-enolase, respectively. Segments 333–369, 410–456, 421–428 and 535–552 of TPO are homologous to segments 282–324, 22–72, 289–296 and 169–186 of AKRIAI, respectively. Segment 492–566 of TPO is homologous to segment 10–77 of DDAHI [25].

Making reference to Table 2 as an example for describing the other two Tables (Table 1 and Table 3), there are proteins with a single segment of homology each, such as butyrylcholinesterase (aa 9–527 matching aa 2204–2722 of Tg), and other proteins with multiple segments of homology (which are listed from the most N-terminal to the most C-terminal position). Examples of this multiplicity are the nine segments of SPARC-1/SMOC-1 that are homologous to Tg. Close inspection of these nine segments (Table 2) shows that they fall within the long region 54–340 of SPARC-1/SMOC-1, which matches a discontinuous and much longer region of Tg comprised between aa 34 and 1212. Indeed, two long stretches of Tg (aa 359–608 and 662–880) did not match any segment of aa 54–340 of SPARC-1/SMOC-1. The extent of amino acid identity with Tg segments ranges from 22% (KIAA1366 protein) to 50% (aa 333–372 of testican-1 and aa 333–374 of testican-2), and overall homology from 36% (aa 636–752 of signal peptide, CUB and EGF-like domain-containing protein 1) to 67% (aa 333–372 of testican-1). Of interest, the group of Tg segments homologous to CNS-expressed proteins (Table 2) and the group of Tg segments homologous to alpha-enolase, AKRIAI or DDAHI [25] showed several overlaps. In detail, Tg segments of the first group fully contained a Tg segment of the second group in 59 cases (with some multiple matches) and were fully contained in a Tg segment of the second group in 3 cases, while a partial overlap of more than 10 residues was observed in 10 cases.

This pattern of proteins having a single segment of homology (for instance, protocadherin Fat 4) or other proteins having multiple segments of homology (for instance, fibrillin-1 and fibrillin-3) applied to TPO (Table 3). Identity with TPO ranges from 22% (prostaglandin G/H synthase 1/cyclooxygenase-1) to 54% (aa 158–191 of Adhesion G protein-coupled receptor E2/EGF-like module receptor 2/CD312), and overall homology from 36% (aa 1621–1706 of LTBP-1) to 64% (thrombospondin-3 and aa 2204–2236 of fibrillin-3). TPO segments homologous to CNS-expressed proteins (Table 3) fully contained a Tg segment homologous to alpha-enolase, AKRIAI or DDAHI [25] in 36 cases (with many multiple matches), and two partial overlaps of more than 10 residues were also observed.

In the case of TSH-R (Table 1), with the only exception of LGR4, all proteins (which were cell receptors, except chondroadherin) had a single segment of homology with the thyroid autoantigen. Identity with TSH-R ranges from 19% (probable G-protein coupled receptor 34, alpha-1B adrenergic receptor and bombesin receptor subtype-3) to 55% (G protein-coupled receptor), and overall homology from 36% (Melanopsin/Opsin-4 and 5-hydroxytryptamine receptor 7) to 75 % (G protein-coupled receptor). TSH-R segments homologous to CNS-expressed proteins (Table 1) fully contained a Tg segment homologous to alpha-enolase, AKRIAI or DDAHI [25] in 122 cases (with many multiple matches), while the partial overlaps of more than 10 residues were five.

### Topographic position of the homologous proteins with respect to domains and epitopic regions of each thyroid autoantigen

**Fig. 1:**
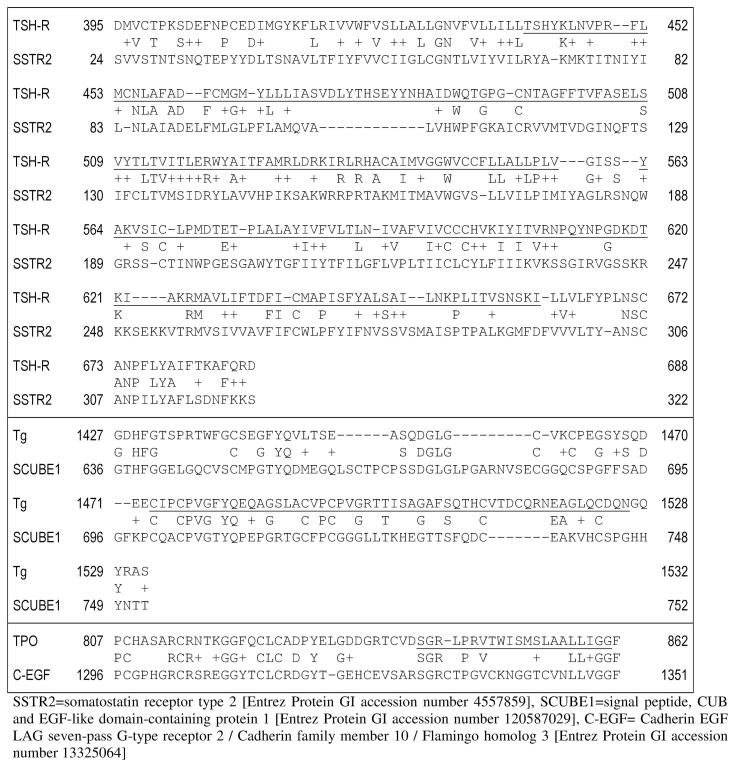
Illustrative examples of amino acid sequence homologies between CNS proteins and TSH-R, Tg and TPO (top, middle and bottom panel, respectively). Epitopes of the three thyroid autoantigens are underlined.

Fig. 1 provides illustrative examples for TSH-R, Tg and TPO (top, middle and bottom panel, respectively), with their epitopes highlighted.

**Fig. 2:**
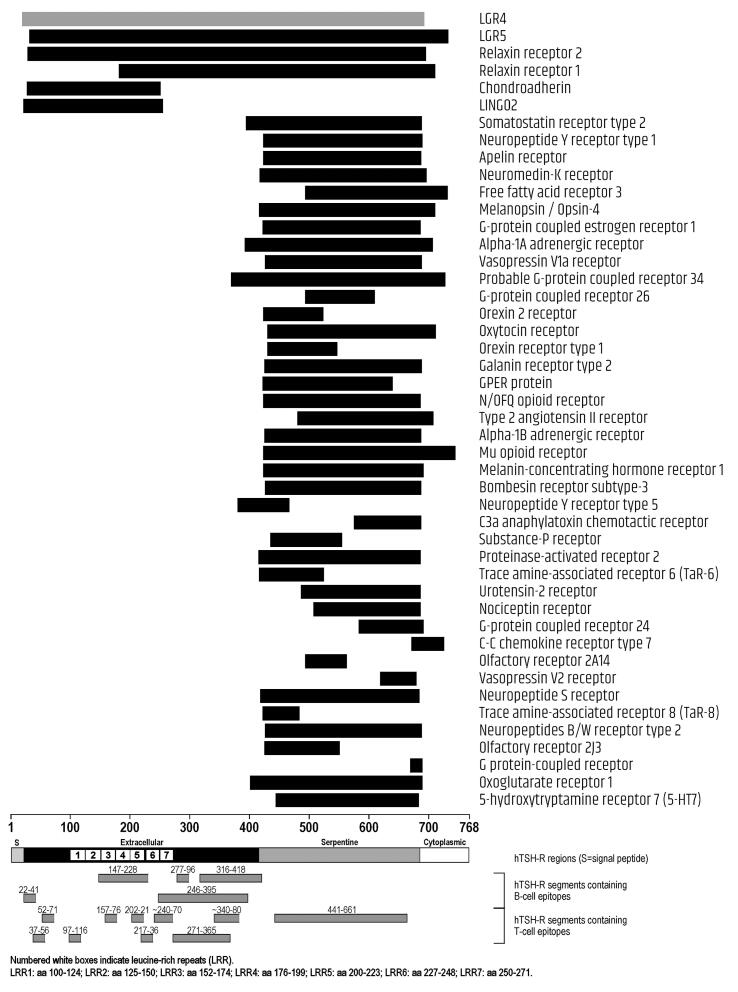
Homologies between CNS-expressed proteins and TSH-R. Segments in black represent single homologous sequences, segments in gray represent the cumulative span of multiple, overlapping homologous sequences of the same protein.

**Fig. 3:**
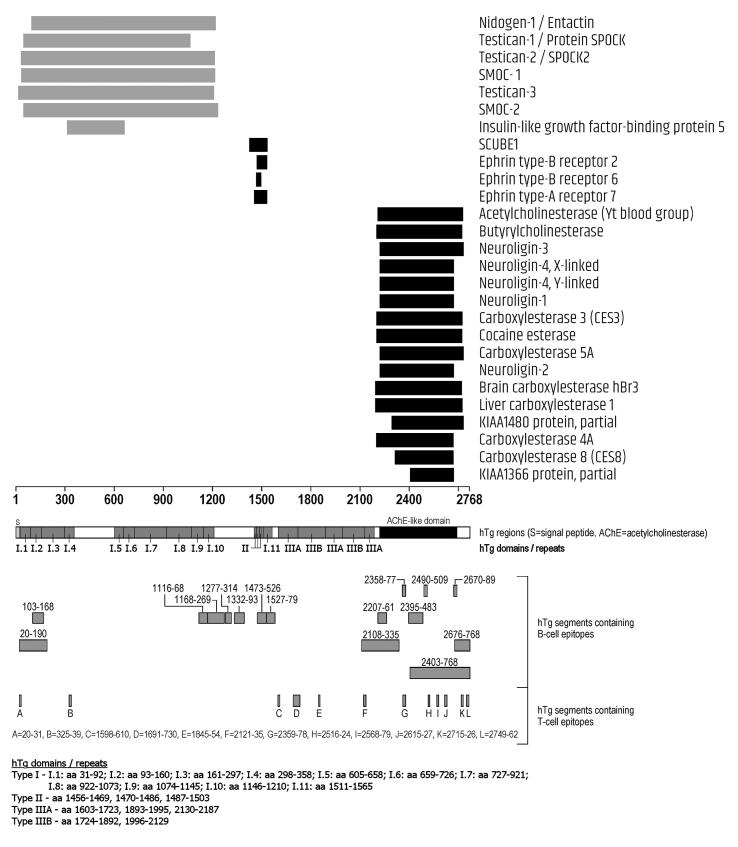
Homologies between CNS-expressed proteins and Tg. Segments in black represent single homologous sequences, segments in gray represent the cumulative span of multiple, overlapping homologous sequences of the same protein.

**Fig. 4:**
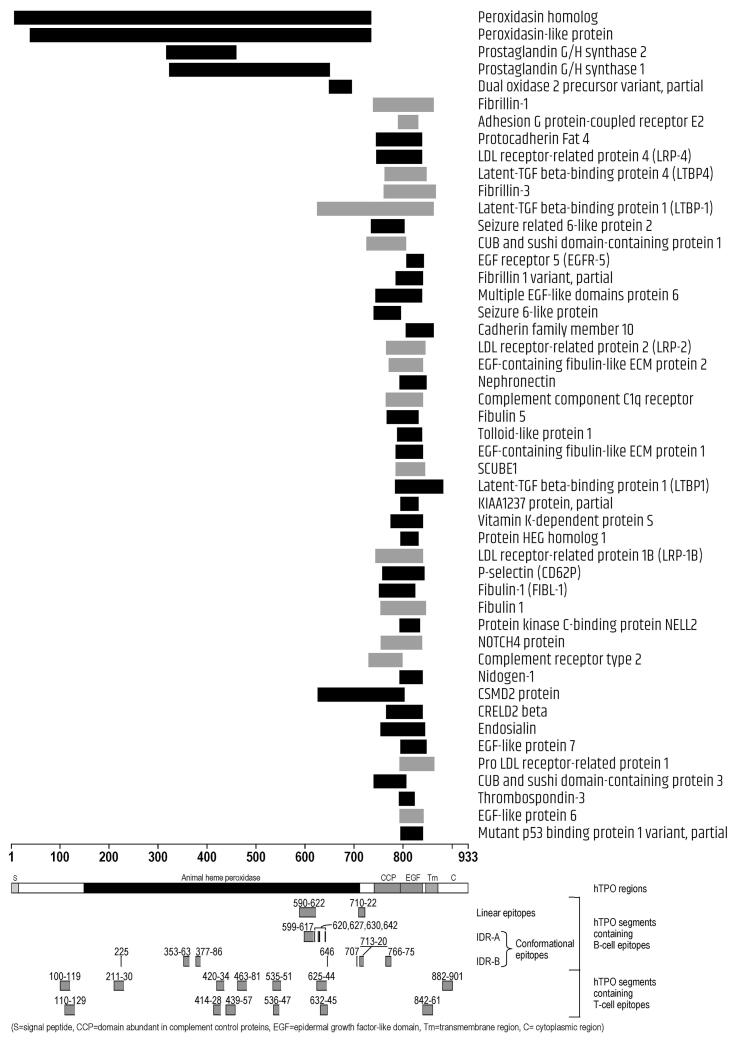
Homologies between CNS-expressed proteins and TPO. Segments in black represent single homologous sequences, segments in gray represent the cumulative span of multiple, overlapping homologous sequences of the same protein.

The position of sequence homology within given domains of the three thyroid autoantigens can be appreciated in Fig. 2, Fig. 3, Fig. 4. Of the 46 proteins homologous to TSH-R (Fig. 2), only the first 6 (LGR4, LGR5, relaxin receptor 1, relaxin receptor 2, chondroadherin and LINGO2) match the whole length of TSH-R, while the others match the serpentine domain, most frequently for its whole length. A few proteins match the C-terminus of the extracellular domain, and a few match the intracellular domain. With the single exception of G protein-coupled receptor and C–C chemokine receptor type 7 (whose homology with TSH-R starts at aa 670 and 672, respectively, of the thyroid autoantigen), all other 44 proteins matched TSH-R regions containing at least one epitope (Fig. 2).

Concerning Tg (Fig. 3), of the 27 homologous proteins, 7 matched a long N-terminal region, 4 a very short central region, and the remaining 11 the acethylcolinesterase-like domain at the C-terminus of Tg. Noteworthy, all 27 proteins matched regions of Tg containing at least one epitope, including the short Tg segment 1470–1494 matched by Ephrin type-B receptor 6, since the aa sequence 1473–1526 of Tg is epitopic (Fig. 3).

Concerning TPO (Fig. 4), of the 48 homologous proteins, 2 (peroxidasin homolog, and peroxidasin-like protein) matched the long whole heme-peroxidase domain (residues 142–738) and the N-terminal segment ahead of it, 3 matched part of the heme-peroxidase domain (prostaglandin G/H synthase 1, prostaglandin G/H synthase 2, and dual oxidase 2 precursor variant), while the remaining 43 matched the complement control protein-like domain (CCP-like domain at residues 740–795) and/or the epidermal growth factor (EGF)-like domain (EGF-like domain, residues 796–846), with a few matching also the end of the heme-peroxidase and a few matching part of the transmembrane domain (residues 847–871). Noteworthy, one CNS-protein (nidogen-1/entactin), shared homology also with Tg. The segment 800–840 of nidogen-1/entactin was 43% identical and 50% homologous to the segment 794–839 of TPO (Table 3 and Fig. 4). On the other hand, 6 segments of nidogen-1/entactin spanning aa 847–925 were 35–45% identical and 50–58% homologous to six segments of Tg: 96–161, 315–358, 660–726, 882–921, 1015–1076 and 1159–1216 (Table 2 and Fig. 3). While the segment 794–839 of TPO, and 315–358, 660–726, 882–921 and 1015–1076 of Tg do not contain epitopes, the Tg segment 96–161 and 1159–1216 contain epitopes at aa 20–190, 1116–1168 and 1168–1269 (Fig. 3 and Fig. 4).

For 18 of the proteins shown in Table 3, all homologies were with segments of TPO which do not contain epitopes or had 6 or less amino acids of overlap with TPO epitopes. These proteins were, in alphabetical order: adhesion G protein-coupled receptor E2, C-type lectin domain family 14 member A, dual oxidase 2 precursor variant, EGF-containing fibulin-like extracellular matrix protein 1, EGF-containing fibulin-like extracellular matrix protein 2, EGF-like protein 6, EGF-like protein 7, fibrillin 1 variant, KIAA1237 protein, mutant p53 binding protein 1, nephronectin, nidogen-1, protein HEG homolog 1, protein kinase C-binding protein NELL2, signal peptide, CUB and EGF-like domain-containing protein 1, thrombospondin-3, tolloid-like protein 1, vitamin K-dependent protein S. For 16 other proteins, homologies included only segments belonging to IDR-B: this was the case of fibrillin-1, protocadherin Fat 4, low-density lipoprotein receptor-related protein 4, latent-transforming growth factor beta-binding protein 4, seizure related 6-like protein 2, CUB and sushi domain-containing protein 1, multiple epidermal growth factor-like domains protein 6, seizure 6-like protein, complement component C1q receptor, low-density lipoprotein receptor-related protein 1B, P-selectin, fibulin-1, NOTCH4 protein, complement receptor type 2, endosialin, CUB and sushi domain-containing protein 3.

### The thyroid-autoantigen-homologous proteins are expressed in given areas of the CNS, and almost all of them are expressed in the thyroid

**Supplementary**Tables 1–3 summarize information from the Expression Atlas (https://www.ebi.ac.uk/gxa/home) [37] about the expression of the proteins homologous to TSH-R, Tg and TPO, respectively, in different areas of the CNS and in the thyroid.

The same Supplementary Tables also show, highlighted in gray, which areas of CNS expressing thyroid autoantigen-homologous proteins were found to show abnormalities at diagnostic neuroimaging in patients with HE/SREAT (references about these data are available upon request). Of these areas, those with the highest number of TSH-R-homologous proteins expressed were frontal lobe (n = 36), cerebral cortex (n = 34), frontal cortex and temporal lobe (n = 33 each); those with the highest number of Tg-homologous proteins expressed were frontal lobe and temporal lobe (n = 26 each) followed by brain, cerebral cortex and frontal cortex (n = 25 each); those with the highest number of TPO-homologous proteins expressed were brain (n = 47), temporal lobe (n = 45), cerebral cortex, frontal cortex and frontal lobe (n = 44 each).

For a few proteins homologous to TSH-R (free fatty acid receptor 3, trace amine-associated receptor 6, olfactory receptor 2A14, trace amine-associated receptor 8, olfactory receptor 2 J3), the Expression Atlas provides no details on which CNS areas express these proteins. Also for a few proteins, the same Atlas provides no details as to whether the thyroid gland expresses these proteins, or reports that their expression is below the cutoff value considered (**Supplementary** Tables 1–3). **Supplementary** Table 4 shows data reported in the Expression Atlas about the expression of the three currently known autoantigens of HE/SREAT in the thyroid and in the brain/CNS.

### Autoantibodies against the thyroid-autoantigen-homologous CNS-expressed proteins have been detected in a number of autoimmune diseases

As explained under Materials and Methods, we probed the literature for articles on the presence of serum autoantibodies against each of the thyroid autoantigen-homologous proteins in autoimmune diseases, including thyroid autoimmune diseases, by performing a PubMed search with the string “(autoanti* OR autoimm* OR autoreact*) AND” followed by the name of each protein and manually selecting relevant original papers [38], [39], [40], [41], [42], [43], [44], [45], [46], [47], [48], [49], [50], [51], [52], [53], [54], [55], [56], [57], [58], [59], [60], [61], [62], [63], [64], [65], [66], [67], [68], [69], [70], [71], [72], [73], [74], [75], [76], [77], [78], [79], [80], [81], [82], [83], [84], [85], [86], [87], [88], [89], [90], [91], [92], [93]. As summarized in Table 4, of the 46 CNS proteins homologous to TSH-R, 5 (11%; LGR4, chondroadherin, alpha-1A adrenergic receptor, Mu opioid receptor, and melanin-concentrating hormone receptor 1) were reported to stimulate autoAb, and in the following conditions: CNS demyelinating disease, autoimmune hepatitis, refractory hypertension, psychiatric disorders, chronic fatigue syndrome and vitiligo [38], [39], [40], [41], [42], [43], [44], [45], [46], [47], [48], [49], [50], [51]. Of the utmost interest are anti-LGR4 autoAbs, because they were detected also in patients with AIT [30]. Noteworthy is also information available on epitopes of melanin-concentrating hormone receptor 1 [27], with aa 85–98 and 254–260 being major autoantibody epitopes, aa 51–80 and 154–158 being minor autoantibody epitopes, and aa 254–260 being the target of function-blocking antibodies. Thus, the segment 119–393 of melanin-concentrating hormone receptor 1, which we found to be homologous to the segment 424–691 of TSH-R contains epitopes (aa 154–158 and 254–260), as does the homologous TSH-R segment (epitope at aa 441–661).

**Table 4:** Involvement in autoimmune disorders, as resulting from a PubMed search, of the proteins that we found share local homology with thyrotropin receptor (TSH-R).

**Protein**	**No. of articles**	**Citations**	**Results**
Leucine-rich repeat-containing G-protein coupled receptor 4 (LGR4)	1	Greer JM et al. 2017 [38]	Patients with both CNS disease and AITD have elevated levels of T cells and antibodies to LGR4, which is expressed in brainstem and spinal cord
Chondroadherin	1	Mazzara S et al. 2015 [39]	Autoantibodies to chondroadherin are present in autoimmune hepatitis patients and could be used as diagnostic/prognostic markers
Alpha-1A adrenergic receptor	2	Wenzel K et al. 2008 [40]	Agonistic autoantibodies to alpha-1A adrenergic receptor are present in patients with hypertension and are a possible cause of hypertension.
		Wenzel K et al. 2010 [41]	In a rat model, autoantibodies to alpha-1A adrenergic receptor may contribute to cardiovascular damage.
Mu opioid receptor	5	Tanaka S et al. 2003 [42]	Autoantibodies to mu opioid receptor were found in 13.1% of 122 psychiatric patients.
		Tanaka S et al. 2003 [43]	Autoantibodies to mu opioid receptor were found in 15.2% of 60 patients with chronic fatigue syndrome
		Macé G et al. 2002 [44]	Autoantibodies to mu opioid receptor are commonly expressed in healthy humans and may promote Fas-mediated apoptosis
		Macé G et al. 1999 [45]	Autoantibodies that bind the first and third extracellular loops of the mu opioid receptor mimic morphine-induced receptor activation
		Macé G et al. 1999 [46]	Some IgG autoantibodies to mu opioid receptor have a morphine-like activity
Melanin-concentrating hormone receptor 1	5	Kroon MW et al. 2013 [47]	Autoantibodies to melanin-concentrating hormone receptor 1 are common in the sera of patients with vitiligo
		Li Q et al. 2011 [48]	Melanin-concentrating hormone receptor 1 is a well-known autoantigen in vitiligo
		Gavalas NG et al. 2009 [49]	In vitiligo patients, peptides 85–98 and 254–260 are major autoantibody epitopes of melanin-concentrating hormone receptor 1, peptides 51–80 and 154–158 are minor autoantibody epitopes, peptide 254–260 is the target of function-blocking antibodies.
		Gottumukkala RV et al. 2003 [50]	Several domains of melanin-concentrating hormone receptor 1 are recognized by autoantibodies from vitiligo patients.
		Kemp et al. 2002 [51]	Melanin-concentrating hormone receptor 1 is an autoantigen in vitiligo

Of the 46 TSH-R homologous proteins, 41 do not appear in the Table, because we retrieved no literature about their involvement in autoimmune disorders. These proteins are: Leucine-rich repeat-containing G-protein coupled receptor 5 (LGR5), Relaxin receptor 2/Leucine-rich repeat-containing G-protein coupled receptor 8 (LGR8), Relaxin receptor 1/Leucine-rich repeat-containing G protein-coupled receptor 7 (LGR7), Leucine-rich repeat and immunoglobulin-like domain-containing nogo receptor-interacting protein 2 (LINGO2), Somatostatin receptor type 2, Neuropeptide Y receptor type 1, Apelin receptor, Neuromedin-K receptor/Neurokinin B receptor/Tachykinin receptor 3, Free fatty acid receptor 3, Melanopsin/Opsin-4, G-protein coupled estrogen receptor 1/Membrane estrogen receptor, Vasopressin V1a receptor, Probable G-protein coupled receptor 34, G-protein coupled receptor 26, Orexin 2 receptor, Oxytocin receptor, Orexin receptor type 1, Galanin receptor type 2, GPER protein, N/OFQ opioid receptor, Type 2 angiotensin II receptor, Alpha-1B adrenergic receptor, Bombesin receptor subtype-3, Neuropeptide Y receptor type 5, C3a anaphylatoxin chemotactic receptor, Substance-P receptor/Tachykinin receptor 1, Proteinase-activated receptor 2, Trace amine-associated receptor 6 (TaR-6), Urotensin-2 receptor/G-protein coupled receptor 14, Nociceptin receptor, G-protein coupled receptor 24, C–C chemokine receptor type 7/Epstein-Barr virus-induced G-protein coupled receptor 1/MIP-3 beta receptor, Olfactory receptor 2A14, Vasopressin V2 receptor, Neuropeptide S receptor, Trace amine-associated receptor 8 (TaR-8), Neuropeptides B/W receptor type 2, Olfactory receptor 2 J3, G protein-coupled receptor, Oxoglutarate (alpha-ketoglutarate) receptor 1, 5-hydroxytryptamine receptor 7 (5-HT7).

As summarized in Table 5, of the 27 CNS proteins homologous to Tg, 2 (7%; nidogen-1/entactin and ephrin type-B receptor 2) were reported to generate autoAb, and in the following conditions: certain types of glomerulonephritis, autoimmune uveoretinitis, systemic lupus erythematosus and related disorders (systemic vasculitis, rheumatoid arthritis), pulmonary renal syndrome, the Aicardi-Goutières syndrome, acute necrotizing encephalopathy, and systemic sclerosis [52], [53], [54], [55], [56], [57], [58], [59], [60], [61], [62]. As mentioned above (see heading “Topographic position of the homologous proteins with respect to domains and epitopic regions of each thyroid autoantigen”) of the 6 Tg-homologous segments of nidogen-1/entactin (of which one matches the epitopic region of Tg at aa 20–190, and another overlaps with the two epitopic region of Tg 1116–1168 and 1168–1269), four entirely contain the epitope 867–887 (segments 849–917, 847–919, 859–922 and 867–925), while two partially overlap with it (segments 874–919 and 880–919). It is noteworthy that segments 849–917 and 867–925 of nidogen-1, which entirely include an epitope of this protein, are homologous to two segments of Tg which correspond to epitopes of this thyroid autoantigen.

**Table 5:** Involvement in autoimmune disorders, as resulting from a PubMed search, of the proteins that we found share local homology with thyroglobulin.

**Protein**	**No. of articles**	**Citations**	**Results**
Nidogen-1/Entactin	9	Fukatsu A et al. 1987 [52]	Rats injected with mercuric chloride develop autoantibodies to various components of the glomerular basement membrane, including emtactin
		Saxena R et al. 1990 [53]	Entactin is a possible autoantigen of the glomerular basement membrane, which could be involved in some types of human autoimmune glomerulonephritis (non-Goodpasture)
		Saxena R et al. 1991 [54]	Anti-entactin antibodies were found in extracapillary glomerulonephritis patients, although very few.
		Saxena R et al. 1991 [55]	Circulating anti-entactin antibodies are present in specific types of glomerulonephritis, but not in others nor in healthy subjects.
		Wang J et al. 1994 [56]	In the iris of rats with experimental autoimmune uveoretinitis, there is an increase in immunoreactivity of several proteins, including entactin
		Saxena R et al. 1994 [57]	Patients with systemic lupus erythematosus often have anti-entactin antibodies, which are more common in case of severe disease.
		Saxena R et al. 1995 [58]	Two of 40 patients with pulmonary renal syndrome had anti-entactin autoantibodies
		Li QZ et al. 2005 [59]	Autoantibodies to entactin are frequent in patients with lupus but not associated with disease activity
		Cuadrado E et al. 2015 [60]	IgG antibodies to several autoantigens, including entactin, are present in patients with Aicardi-Goutières syndrome, an autoimmune disorder with some similarities to systemic lupus erythematous which particularly targets the cerebral white matter.
Ephrin type-B receptor 2	2	Shirai T et al. 2013 [61]	Autoantibodies to ephrin type B receptor 2 were found in a patient with acute necrotizing encephalopathy and systemic lupus erythematosus, but not in patients with lupus only.
		Azzouz DF et al. 2016 [62]	Patients with systemic sclerosis or systemic lupus erythematosus show autoantibodies to ephrin type B receptor 2

Of the 27 Tg homologous proteins, 25 do not appear in the Table, because we retrieved no literature about their involvement in autoimmune disorders. These proteins are: Testican-1/Protein SPOCK, Testican-2/SPARC/osteonectin, CWCV, and Kazal-like domains proteoglycan 2 (SPOCK2), SPARC-related modular calcium-binding protein 1/Secreted modular calcium-binding protein 1 (SMOC-1), Testican-3, SPARC-related modular calcium-binding protein 2/Secreted modular calcium-binding protein 2 (SMOC-2), Insulin-like growth factor-binding protein 5, CUB and EGF-like domain-containing protein 1, Ephrin type-B receptor 6, Ephrin type-B receptor 7, Acetylcholinesterase (Yt blood group), Butyrylcholinesterase, Neuroligin-3, Neuroligin-4, X-linked, Neuroligin-4, Y-linked, Neuroligin-1, Carboxylesterase 3 (CES3), Cocaine esterase, Carboxylesterase 5A, Neuroligin-2, Brain carboxylesterase hBr3, Liver carboxylesterase 1/Acyl-coenzyme A:cholesterol acyltransferase/Brain carboxylesterase hBr1/Cocaine carboxylesterase/Egasyn/Methylumbelliferyl-acetate deacetylase 1/Monocyte/macrophage serine esterase/Retinyl ester hydrolase/Serine esterase 1/Triacylglycerol hydrolase, KIAA1480 protein, Carboxylesterase 4A, Carboxylesterase 8 (CES8), KIAA1366 protein.

Concerning ephrin type-B receptor 2, the only Tg-homologous segment (aa 269–312) marginally overlaps with a known epitope (aa 309–318) of the protein, while its Tg counterpart (aa 1473–1531) entirely contains the Tg epitope 1473–1526.

As summarized in Table 6, of the 47 CNS proteins homologous to TPO, 7 (15%; fibrillin-1/asprosin, fibrillin-3, LRP-2, LRP-4, P-selectin/CD62P/granule membrane protein 140/leukocyte-endothelial cell adhesion molecule 3, and the aforesaid nidogen-1/entactin) were reported to generate autoAb, and in the following conditions: recurrent pregnancy loss, pregnancy-induced hypertension, systemic sclerosis, localized scleroderma, CREST (calcinosis, Raynaud's esophageal dysmotility, sclerodactyly, and telangiectasia) syndrome, mixed connective tissue disease, type 1 diabetes mellitus, primary pulmonary hypertension syndrome, myasthenia gravis, autoimmune polyglandular syndrome type 3, amyotrophic lateral sclerosis, ABBA disease (a renal disease characterized by kidney antibrush border antibodies and renal failure), rheumatoid arthritis, osteoarthritis, systemic lupus erythematosus, Behçet's disease, and idiopathic thrombocytopenic purpura [52], [53], [54], [55], [56], [57], [58], [59], [60], [63], [64], [65], [66], [67], [68], [69], [70], [71], [72], [73], [84], [85], [86], [87], [88], [89], [90], [91], [92], [93]

**Table 6:** Involvement of the proteins that we found share local homology with thyroperoxidase in autoimmune disorders, as resulting from a PubMed search.

**Protein**	**No. of articles**	**Citations**	**Results**
Fibrillin-1/Asprosin/Epididymis secretory sperm binding protein	11	Atanasova MA et al. 2011 [63]	Increased anti-fibrillin-1 IgM antibodies in patients with recurrent pregnancy loss may contribute to the pathogenesis of this condition
		Admou B et al. 2009 [64]	Anti-fibrillin-1 autoantibodies may be present in systemic sclerosis patients
		Grassegger A et al. 2008 [65]	Anti-fibrillin-1 autoantibodies seem to have important roles in the pathogenesis of systemic sclerosis
		Zhou X et al. 2005 [66]	Anti-fibrillin-1 autoantibodies are specifically present in systemic sclerosis patients and may induce activation of normal dermal fibroblasts into a profibrotic phenotype, which resembles that of systemic sclerosis
		Nicoloff G et al. 2005 [67]	Anti-fibrillin-1 autoantibodies can be found in diabetic patients
		Pandey JP et al. 2001 [68]	Anti-fibrillin-1 autoantibodies in systemic sclerosis patients are associated with specific KM and GM allotypes (genetic markers of immunoglobulin kappa and gamma chains, respectively)
		Tan FK et al. 2000 [69]	Anti-fibrillin-1 autoantibodies in systemic sclerosis patients correlate with specific ethnic groups but not HLA alleles
		Morse JH et al. 2000 [70]	Anti-fibrillin-1 autoantibodies are present in primary pulmonary hypertension, other than in systemic sclerosis, CREST (calcinosis, Raynaud's esophageal dysmotility, sclerodactyly, and telangiectasia) syndrome, mixed connective tissue disease.
		Lundberg I et al. 2000 [71]	Anti-fibrillin-1 autoantibodies are present in CREST (calcinosis, Raynaud's esophageal dysmotility, sclerodactyly, and telangiectasia) syndrome and mixed connective tissue disease.
		Arnett FC et al. 1999 [72]	Anti-fibrillin-1 autoantibodies are present in patients with linear scleroderma or morphea.
		Tan FK et al. 1999 [73]	Anti-fibrillin-1 autoantibodies may be found in patients with systemic sclerosis, CREST (calcinosis, Raynaud's esophageal dysmotility, sclerodactyly, and telangiectasia) syndrome or mixed connective tissue disease.
Low-density lipoprotein receptor-related protein 4 (LRP-4)	12	Inoue H et al. 2020 [74]	Case report of a patient affected by myasthenia gravis and autoimmune polyglandular syndrome type 3, with autoantibodies to both acetylcholine receptor and low-density lipoprotein receptor-related protein 4 antibody
		Park KH et al. 2018 [75]	Analysis of multiple autoantibodies (including those to low-density lipoprotein receptor-related protein 4) in patients with myasthenia gravis
		Ohnari K et al. 2018 [76]	Report of a case of myasthenia gravis and amyotrophic lateral sclerosis, with autoantibodies to acetylcholine receptor and low-density lipoprotein receptor-related protein 4
		Kruger JM et al. 2017 [77]	Report of a case of myasthenia gravis with autoantibodies to low-density lipoprotein receptor-related protein 4, but not to acetylcholine receptor nor to muscle-specific kinase
		Ishikawa H et al. 2017 [78]	Report of two cases of myasthenia gravis and invasive thymoma, with autoantibodies to acetylcholine receptor and low-density lipoprotein receptor-related protein 4
		Li Y et al. 2017 [79]	Identification of autoantibodies to low-density lipoprotein receptor-related protein 4 in Chinese patients with myasthenia gravis
		Takahashi H et al. 2016 [80]	Report of two cases of amyotrophic lateral sclerosis with autoantibodies to low-density lipoprotein receptor-related protein 4, who showed myasthenic symptoms
		Marino M et al. 2015 [81]	Analysis of the presence of autoantibodies to low-density lipoprotein receptor-related protein 4 in an Italian cohort of 101 myasthenic patients, 45 healthy blood donors and 40 patients with other neurological diseases
		Zisimopoulou P et al. 2014 [82]	Autoantibodies to low-density lipoprotein receptor-related protein 4 were found in 18.7% of about 800 patients with myasthenia gravis from 10 countries
		Zouvelou V et al. 2013 [83]	Report of two cases of myasthenia gravis with autoantibodies to low-density lipoprotein receptor-related protein 4, but not to acetylcholine receptor nor to muscle-specific kinase
		Motomura M et al. 2012 [84]	Autoantibodies to low-density lipoprotein receptor-related protein 4 were found in 9/300 patients with generalized myasthenia gravis negative for anti- acetylcholine receptor autoantibodies
		Higuchi O et al. 2011 [85]	First report of the presence and pathogenetic role of autoantibodies to low-density lipoprotein receptor-related protein 4 in patients with myasthenia gravis
Fibrillin-3	1	Dolcino M et al. 2014 [86]	The peptide TNRRGRGSPGAL, recognized by nearly all sera of patients with psoriatic arthritis, shows amino acid sequence homology and cross-reacts with some skin autoantigens, including fibrillin-3.
fibrillin 1 variant, partial	11	Atanasova MA et al. 2011 [63]	Increased anti-fibrillin-1 IgM antibodies in patients with recurrent pregnancy loss may contribute to the pathogenesis of this condition
		Admou B et al. 2009 [64]	Anti-fibrillin-1 autoantibodies may be present in systemic sclerosis patients
		Grassegger A et al. 2008 [65]	Anti-fibrillin-1 autoantibodies seem to have important roles in the pathogenesis of systemic sclerosis
		Zhou X et al. 2005 [66]	Anti-fibrillin-1 autoantibodies are specifically present in systemic sclerosis patients and may induce activation of normal dermal fibroblasts into a profibrotic phenotype, which resembles that of systemic sclerosis
		Nicoloff G et al. 2005 [67]	Anti-fibrillin-1 autoantibodies can be found in diabetic patients
		Pandey JP et al. 2001 [68]	Anti-fibrillin-1 autoantibodies in systemic sclerosis patients are associated with specific KM and GM allotypes (genetic markers of immunoglobulin kappa and gamma chains, respectively)
		Tan FK et al. 2000 [69]	Anti-fibrillin-1 autoantibodies in systemic sclerosis patients correlate with specific ethnic groups but not HLA alleles
		Morse JH et al. 2000 [70]	Anti-fibrillin-1 autoantibodies are present in primary pulmonary hypertension, other than in systemic sclerosis, CREST (calcinosis, Raynaud's esophageal dysmotility, sclerodactyly, and telangiectasia) syndrome, mixed connective tissue disease.
		Lundberg I et al. 2000 [71]	Anti-fibrillin-1 autoantibodies are present in CREST (calcinosis, Raynaud's esophageal dysmotility, sclerodactyly, and telangiectasia) syndrome and mixed connective tissue disease.
		Arnett FC et al. 1999 [72]	Anti-fibrillin-1 autoantibodies are present in patients with linear scleroderma or morphea.
		Tan FK et al. 1999 [73]	Anti-fibrillin-1 autoantibodies may be found in patients with systemic sclerosis, CREST (calcinosis, Raynaud's esophageal dysmotility, sclerodactyly, and telangiectasia) syndrome or mixed connective tissue disease.
Low-density lipoprotein receptor-related protein 2 (LRP-2)	5	Larsen CP et al. 2018 [87]	Autoantibodies to low-density lipoprotein receptor-related protein 2 can be found in patients with ABBA disease, a kidney disease characterized by kidney antibrush border antibodies and renal failure.
		Ooka S et al. 2003 [88]	Autoantibodies to low-density lipoprotein receptor-related protein 2 were found in patients with rheumatoid arthritis (87%), systemic lupus erythematosus (40%), systemic sclerosis (35%), osteoarthritis (15%), Behçet's disease (3%)
		Dinesh KP et al. 2019 [89]	Report of a case of anti-LRP2 nephropathy/anti-brush border antibody disease
		Yu X et al. 2001 [90]	Detection of amino acid sequence homology and cross-reactivity between CD69 and low-density lipoprotein receptor-related protein 2
		Illies F et al. 2004 [91]	Report of a patient with autoimmune thyroiditis and membranous nephropathy; low-density lipoprotein receptor-related protein 2 (megalin) is expressed on thyroid cells in a TSH-dependent manner and could be a link between the two diseases
P-selectin (CD62P)/Granule membrane protein 140/Leukocyte-endothelial cell adhesion molecule 3/Platelet activation dependent granule-external membrane protein	2	Jiang H et al. 1993 [92]	Autoantibodies to granule membrane protein 140 were found in 13/46 patients with severe pregnancy-induced hypertension
		Zhang S et al. 1995 [93]	Autoantibodies to granule membrane protein 140 were found in 17/92 patients with idiopathic thrombocytopenic purpura
Nidogen-1/Entactin	9	Fukatsu A et al. 1987 [52]	Rats injected with mercuric chloride develop autoantibodies to various components of the glomerular basement membrane, including emtactin
		Saxena R et al. 1990 [53]	Entactin is a possible autoantigen of the glomerular basement membrane, which could be involved in some types of human autoimmune glomerulonephritis (non-Goodpasture)
		Saxena R et al. 1991 [54]	Anti-entactin antibodies were found in extracapillary glomerulonephritis patients, although very few.
		Saxena R et al. 1991 [55]	Circulating anti-entactin antibodies are present in specific types of glomerulonephritis, but not in others nor in healthy subjects.
		Wang J et al. 1994 [56]	In the iris of rats with experimental autoimmune uveoretinitis, there is an increase in immunoreactivity of several proteins, including entactin
		Saxena R et al. 1994 [57]	Patients with systemic lupus erythematosus often have anti-entactin antibodies, which are more common in case of severe disease.
		Saxena R et al. 1995 [58]	Two of 40 patients with pulmonary renal syndrome had anti-entactin autoantibodies
		Li QZ et al. 2005 [59]	Autoantibodies to entactin are frequent in patients with lupus but not associated with disease activity
		Cuadrado E et al. 2015 [60]	IgG antibodies to several autoantigens, including entactin, are present in patients with Aicardi-Goutières syndrome, an autoimmune disorder with some similarities to systemic lupus erythematous which particularly targets the cerebral white matter.

Of the 47 TPO homologous proteins, 40 do not appear in the Table, because we retrieved no literature about their involvement in autoimmune disorders. These proteins are: Peroxidasin homolog/Melanoma-associated antigen MG50/Vascular peroxidase 1, Peroxidasin-like protein, Prostaglandin G/H synthase 2/Cyclooxygenase-2, Prostaglandin G/H synthase 1/Cyclooxygenase-1, Adhesion G protein-coupled receptor E2/EGF-like module receptor 2/CD312, Protocadherin Fat 4, Latent-transforming growth factor beta-binding protein 4 (LTBP4), Latent-transforming growth factor beta-binding protein 1 (LTBP-1), Seizure related 6-like protein 2, CUB and sushi domain-containing protein 1, C-type lectin domain family 14 member A/Epidermal growth factor receptor 5 (EGFR-5), Multiple epidermal growth factor-like domains protein 6, Seizure 6-like protein/KIAA0927 protein, Cadherin EGF LAG seven-pass G-type receptor 2/Cadherin family member 10/Flamingo homolog 3, EGF-containing fibulin-like extracellular matrix protein 2, Nephronectin/Preosteoblast EGF-like repeat protein with MAM domain/EGFL6-like, Complement component C1q receptor/CD93, Fibulin 5, Tolloid-like protein 1, EGF-containing fibulin-like extracellular matrix protein 1, Signal peptide, CUB and EGF-like domain-containing protein 1, Latent-transforming growth factor beta-binding protein 1 (LTBP1), KIAA1237 protein, partial, Vitamin K-dependent protein S, Protein HEG homolog 1, Low-density lipoprotein receptor-related protein 1B (LRP-1B), Fibulin-1, Fibulin 1, Protein kinase C-binding protein NELL2, NOTCH4 protein, complement receptor type 2, dual oxidase 2 precursor variant, partial, CSMD2 protein, Cysteine-rich with EGF-like Domains 2 (CRELD2) beta, Endosialin/CD248, Prolow-density lipoprotein receptor-related protein 1/Alpha-2-macroglobulin receptor/Apolipoprotein E receptor/CD91, CUB and sushi domain-containing protein 3, Thrombospondin-3, Epidermal growth factor-like protein 6, Mutant p53 binding protein 1 variant, partial.

Of interest, it was found that the random peptide TNRRGRGSPGAL, which Dolcino et al. found to be recognized by nearly all sera of patients with psoriatic arthritis, shows amino acid sequence homology and cross-reacts with some skin autoantigens, including fibrillin-3 [86]. Of the 22 TPO-homologous segments of fibrillin-1, seven contained, or had some overlap with, an epitope of the protein. In the majority of cases, the autoantigenic peptide reported in literature had some modifications (citrullinated, methylated or cysteinylated). In detail, segment 723–765 contained the epitopes 733–748 (citrullinated in R11) and 737–752 (citrullinated in R7), segment 908–952 contained the epitopes 917–932 (citrullinated in R14), 921–936 (citrullinated in R10), 925–940 (citrullinated in R6) and almost all of the epitope 947–955 (cysteinylated in C4), segment 1174–1238 contained the epitope 1186–1194 and the epitope 1203–1211 (which is reported in literature in two versions, without or with methylation in C6), segment 1216–1280 contained the epitope 1256–1264 (methylated in C8), segment 2289–2334 contained the epitopes 2301–2316 (citrullinated in R6 and R11), 2305–2320 (citrullinated in R2 and R7) and 2309–2324 (citrullinated in R3); segments 1645–1692 and 2413–2507 had rather limited overlap with epitopes 1689–1697 (methylated in C7) and 2502–2510 (methylated in C8), respectively. All parts of TPO homologous to fibrosin-1 had insignificant (5 aa or less) or no overlap with known TPO epitopes, with one exception (segment 740–832, matching aa 2129–2199 of fibrosin-1, contains the entire TPO epitope 766–775 and part of epitope 842–861).

The 17 TPO-homologous segments of fibrillin-3 contained an epitope in four cases, while their TPO counterparts had four complete and one partial overlap with an epitope. The only match between two epitope-containing segments was that between aa 2368–2468 of fibrillin-3 (which include the epitope 2425–2440, citrullinated in R9, and 2429–2444, citrullinated in R5 and R14) and aa 762–782 of TPO (which include the epitope 766–775). The segment 763–816 of fibrillin-3 (which contains the epitope 773–786) matched segment 793–852 of TPO, which has an 11-residue overlap with the autoepitope 842–861 of the protein. The epitope-containing segments without an epitope-containing homolog were localized at positions 570–614 (containing epitope 594–602) and 867–911 (containing epitope 878–886) of fibrillin-3, and positions 794–866, 795–862 (both containing epitope 842–861) and 766–831 (containing epitope 766–775) of TPO. All other homologous segments of both proteins had insignificant or no overlap with known epitopes.

Concerning LRP-2, three TPO-homologous segments were found, of which only one (aa 1388–1428) contained autoepitopes (aa 1397–1412, citrullinated in R12 and R16, and aa 1401–1416, citrullinated in R8 and R12); their TPO counterparts had insignificant or no overlap with known epitopes. Upon describing one patient with AIT and membranous nephropathy, the authors report that low-density lipoprotein receptor-related protein 2 (megalin) is expressed on thyroid cells in a TSH-dependent manner and could be the link between the two diseases [91].

The single local homology found between LRP-4 and TPO involved aa 359–433 of LRP-4, which contain the epitopes 361–376 (citrullinated in R13) and 365–380 (citrullinated in R9), and aa 747–838 of TPO, which contain the epitope 766–775.

A single local homology was found also between P-selectin and TPO, but in this case neither segment (aa 531–621 and 759–843, respectively) contained epitopes (there was only an overlap of few residues in the case of the TPO segment).

## Discussion

Expanding our previous data [25], we have provided some evidence for molecular mimicry between thyroid autoantigens and CNS-expressed proteins being a reasonable mechanism for HE/SREAT. First, a limited number of CNS-expressed proteins match relatively short to relatively long sequences of the thyroid autoantigens. Second, the homologous sequences of the three thyroid autoantigens almost always contain at least one epitope. Third, the CNS areas where the thyroid-autoantigen homologous proteins are expressed match CNS areas where abnormalities were detected at biopsy/necropsy and/or by neuroimaging in patients with HE/SREAT. Fourth, the literature associated a number of the homologous CNS-expressed proteins with a number of autoimmune disorders (not necessarily CNS-restricted), in which corresponding serum autoAb were detected.

TSH-R belongs to the superfamily of the rhodopsin-like G protein-coupled receptors (GPCR), whose ectodomain belongs, in turn, to the family of proteins with leucine-rich repeats (LRR) [94]. Thus, many of the homologies found (Table 1, Fig. 2) were not unexpected. Interestingly, the TSH-R regions of homology involve its nine LRR repeats, the serpentine domain (aa 414–682, with seven transmembrane helices) and most of the cytoplasmic tail (aa 683–764). Further to the last 20 residues (aa 745–764), two other TSH-R regions are spared by homologies: the signal peptide (first 20 residues) and, upon ignoring LGR4, LGR5, LGR7 and LGR8, the region 255–369. This last region encompasses the LRR9 repeat at 250–271 and most of the hinge region (aa 272–413) with its TSH-R specific sequence at aa 317–366. This segment 317–366 (also called the 50-residue long C-peptide of TSH-R), that is deleted following an intramolecular cleavage, is TSH-R specific because it is absent in the cognate gonadotropin receptors (FSH-R, LH-R) [95].

Assuming that the CNS-expressed TSH-R undergoes the same intramolecular cleavage as the thyrocyte-expressed TSH-R, then the CNS cell will continue to have a cell-attached TSH-R, so called B subunit, with a few extracellular residues distal to the cleaved 317–366 segment, the whole serpentine domain and the intracellular C-terminus. This approximately 400-residue long portion of TSH-R will retain zones of homology with alpha-enolase, AKRIA and several CNS-expressed proteins, as well as a number of epitopes. Most of these epitopes bind TSH-R Ab that inhibit the TSH-R signaling. Thus, it is possible that, whatever the function(s) of TSH-R may be in the CNS, binding to these Ab might inhibit such function(s).

Also not surprising is the presence of esterases in the list of proteins omologous to the C-terminal part of Tg, because the segment starting at aa 2188 is the acetylcholinesterase domain of this thyroid autoantigen. As reported by Veneziani et al. [96] “*type I repeats of Tg share varying degrees of homology with a six-residue cysteine motif found in a variety of proteins. These include: …. the cell-adhesion protein nidogen/entactin, the insulin-like growth factor binding protein (IGFBP), … the proteoglycan testican…*”. Moreover, “*The cysteine-rich units of Tg share limited structural analogy with the epidermal growth factor (EGF-) homologous repeats found, in single or multiple copies, in a variety of proteins… The homology between EGF-like modules is based primarily on the position of six cysteins (numbered Cys1 through CysVI). Type I repeats of Tg differ from typical EGF-like modules for the spacings between some of the cysteines, and the presence of unrelated inserts of variable length at conserved positions”*.

Finally, because TPO belongs to the Haem peroxidase superfamily, namely haem-containing enzymes that use hydrogen peroxide as the electron acceptor to catalyse oxidative reactions (http://www.ebi.ac.uk/interpro/entry/IPR019791), homologies with peroxidasins, prostaglandin G/H synthases/cyclooxygenases, dual oxidase 2 were expected. In addition, the stretch 742–795 of TPO contains SUSHI repeats that have been identified in several proteins of the complement system, while aa 796–838 is the calcium-binding EGF domain (https://www.ncbi.nlm.nih.gov/protein/AAA61217.2). Accordingly, also not unexpected were the homologies with complement component C1q receptor, CUB and sushi domain-containing proteins (including seizure related 6-like proteins), CUB and EGF-like domain-containing proteins (including Tolloid-like protein 1), endosialin/CD248 (a protein with one EGF-like domain and one sushi domain), P-selectin (CD62P)/granule membrane protein 140/leukocyte-endothelial cell adhesion molecule 3/platelet activation dependent granule-external membrane protein (a protein with one EGF-like domain and multiple sushi domains), cysteine-rich with EGF-like domains 2 beta, adhesion G protein-coupled receptor E2/EGF-like module receptor 2, EGF-containing fibulin-like extracellular matrix proteins, nephronectin/preosteoblast EGF-like repeat protein with MAM domain/EGFL6-like, multiple EGF-like domains protein 6, C-type lectin domain family 14 member A/EGF receptor 5 (EGFR-5), and other proteins with multiple EGF-like domains (fibrillins, protocadherin fat 4, low-density lipoprotein receptor-related proteins, latent-transforming growth factor beta-binding proteins, fibulins, protein HEG homolog 1, protein kinase C-binding protein NELL2, NOTCH4, thrombospondin-3, and nidogen-1/entactin). Of note, aa 846–919 of nidogen 1/entactin correspond to the Tg type 1 repeat domain of this sulfated glycoprotein widely distributed in basement membranes and tightly associated with laminin (https://www.uniprot.org/uniprot/P14543).

Among CNS proteins homologous to TPO, low-density lipoprotein receptor-related protein 4 (LRP4) deserves particular attention. LRP4 has a central role in synaptic development and maintenance, and acts as the muscle receptor for neural agrin, propagating the signal to muscular tyrosin kinase receptors (MuSK) for acetylcholine receptors (AChR) clustering at the neuromuscular junction (NMJ), a peripheral cholinergic synapse between motor neurons and skeletal muscle fibers [97]. LRP4 autoantibodies are detected in some patients with myasthenia gravis (MG), and the inhibition of the LRP4-agrin interaction appears to be responsible, at least in part, for their pathogenicity [98]. In a systematic review, autoimmune thyroid disease was the most frequent of 23 associated autoimmune disorders, occurring in 10% of MG patients [99]. LRP4 antibodies have also been detected in 10–23% of amyotrophic lateral sclerosis (ALS) patients [100], [101]

As to the NMJ, neurotransmission in the CNS requires precise control of neurotransmitter release from presynaptic terminals and responsiveness of neurotransmitter receptors on postsynaptic membrane, and this process is regulated by glial cells; however, underlying mechanisms are not fully understood. Being expressed in the brain, LRP4 has been implicated in hippocampal synaptic plasticity [102], [103]. It has been demonstrated that glutamate release in the hippocampal regions of the brain is impaired in LRP4-defective mice, revealing a critical role of the LRP4-agrin signaling in modulating astrocytic ATP release and synaptic glutamatergic transmission [103], [104]. More recently, it has been demonstrated that LRP4 is reduced in the brain of patients with Alzheimer disease (AD), paralleling the reduced levels in an AD mouse model that are associated with exacerbation of cognitive impairment and increases in the amount of amyloid aggregates [105]. Impaired synaptogenesis and altered synaptic transmission at the temporal regions are commonly associated with cognitive disturbances, behavioural alterations, memory reduction. All the above-mentioned disturbances are likewise described in HE/SREAT. Hence, in the light of the of homology between LRP4 and TPO, and considering that presence of LRP4 in temporal areas of the brain has been described, we could speculate on a possible cross reactivity between anti-TPO antibodies and LRP4, explaining the cognitive and behavioural manifestations of HE/SREAT.

The multiform clinical symptomatology of SREAT and its dramatic responsiveness to the corticosteroid therapy (as also supported by the disappearance of abnormalities detected at neuroimaging and electroencephalography, in parallel with a fall both in the serum and CSF levels of the pre-therapy markedly elevated thyroid Ab levels), is better explained by the following scenario. Prior to therapy, elevated levels of thyroid Ab (*viz*. any of TgAb, TPOAb and TSH-R-Ab) would gain access to the CSF through a damaged blood–brain-barrier. Not only, as we explained previously [25], any of these thyroid Ab can attack CNS cells that express the corresponding autoantigen (*viz*. any of Tg, TPO, TSH-R) but it/they may attack cells that express one or more of CNS-expressed proteins described here and previously [25]. A requisite for this last attack and associated Ab binding with at least one of these proteins is that the thyroid Ab has/have been elicited by one or more epitopes contained in regions of the thyroid autoantigen that share homology with such CNS protein(s). As shown in this paper, a given region of a given thyroid autoantigen can share homology with only one, a few or several CNS-expressed proteins. Hence, it would be hard to find two HE/SREAT patients with the same panel of symptoms. Once steroid therapy has knocked-down thyroid Ab levels and thyroid Ab passage into the CNS, then attacks to the above CNS cells would terminate and symptomatology, neuroimaging and electroencephalography abnormalities disappear.

CNS proteins that share a series of homologies with antibodies associated to HE/SREAT have a prevalent distribution in areas correlated with the limbic system and temporal regions in general, as also supported by the literature data on neuroradiological alterations which are prevalent in these regions in HE/SREAT patients (**Supplementary** Table 1, Table 2, Table 3). This could justify some symptoms, such as confusion, behavior and memory disorders, and epilepsy. In our study, homologies are also detectable among some proteins located in the blood–brain barrier (BBB) (i.e. proteins of the Notch families) and HE/SREAT associated antibodies target, determining a BBB damage and suggesting a possible mechanism of brain aggression by autoantibodies and immunocompetent cells.

A similar mechanism could only be suggested also for cerebellar ataxia in HE/SREAT. In fact, the intimate pathological mechanism underlying cerebellar ataxia development in HE/SREAT has already been investigated, however it remains obscure; an impaired presynaptic short-term plasticity between parallel fiber-Purkinje cell transmissions and defective glutamate release have been postulated as potential pathological mechanisms in some patients with HE/SREAT [106], [107].

The spatial position of the homologous segments in relation to cell compartments (extracellular, transmembrane, intracellular) and in the context of the three-dimensional structure of the respective proteins (conformation and chemical characteristics of protein surface, degree of solvent exposure) may be important in the pathogenesis of autoimmune diseases. As a general rule, autoantibodies against extracellular, solvent exposed parts of a molecule are more often directly pathogenetic, while the role of autoimmunity against parts of the molecules that are normally “hidden” from the immune system is less straightforward.

Some authors compared autoimmune conditions characterized by intracellular and extracellular target autoantigens, pointing out differences and possible implications of this difference in clinical, monitoring, diagnostic and therapeutic terms [108]. By analogy, these considerations could be applied to autoimmunity against exposed and hidden parts of molecules, and this aspect, currently unexplored, could be an intriguing line of future research in the field of SREAT.

In conclusion, we support our idea of HE/SREAT being ignited by thyroid autoantigens that, after having gained access to CS, bind to Tg, TPO and TSH-R expressed in cells of the CNS [25] forming immune complexes. In addition to this mechanism, it is well possible that TgAb, TPOAb, TSH-R-Ab may cross-react with CNS-expressed proteins that share local homology with the corresponding thyroid autoantigens. Depending on the prevalent thyroid Ab that forms the immune complex, the homologous protein(s) that cross-react(s), the CNS area(s) and cell(s) expressing such homologous protein(s) and the resulting impairment of its/their function(s), given symptoms will appear, thus explaining the notoriously multiform clinical presentation and neuroradiological abnormalities of HE/SREAT. If one admits that pathogenicity ensues from TgAb, TPOAb, TSH-R-Ab that gain access to the CNS and the necessity of such Ab to be directed against epitopes that are shared with the corresponding CNS-expressed homologous protein(s), then the probability of occurrence of such epitope requisite would be relatively rare. This rarity fits with the knowledge of HE/SREAT being a rare event compared with the high frequency of HT, the most prevalent autoimmune disease.

At the very minimum, we believe that our data will prompt a number of investigations to directly prove the involvement in HE/SREAT of at least some of the CNS-proteins having homology with the thyroid autoantigens. For instance, one straighforward implication is to check serum thyroid Ab (any of TSH-RAb, TgAb, TPOAb) detected in patients with SREAT for cross-reactivity with the corresponding homogous CNS-proteins (TSH-R-homologous, Tg-homologous, TPO-homologous). Another straightforward translational implication of our data is to characterize epitopically serum thyroid autoantibodies in patients with HT and GD (both TgAb and TPOAb in HT, and at least TSH-RAb in GD). If any of these serum Ab recognize epitopes of the corresponding thyroid autoantigen that fall in regions sharing homology with any of the known HT/SREAT autoantigens (alpha-enolase, AKRIA, DDAHI) and/or any of the CNS-expressed proteins we report here, once it/they has/have been proved as autoantigens associated with HT/SREAT, then HT or GD patients can be sorted out in terms of risk for HT/SREAT.

## Funding

This research did not receive any specific grant from funding agencies in the public, commercial, or not-for-profit sectors.

### CRediT authorship contribution statement

**Salvatore Benvenga:** Conceptualization, Methodology, Data curation, Writing – original draft, Writing – review & editing, Supervision. **Alessandro Antonelli:** Validation, Writing – review & editing. **Poupak Fallahi:** Validation, Writing – review & editing. **Carmen Bonanno:** Validation, Data curation, Writing – original draft. **Carmelo Rodolico:** Validation, Data curation, Writing – original draft. **Fabrizio Guarneri:** Formal analysis, Investigation, Writing – original draft, Visualization.

## Declaration of Competing Interest

The authors declare that they have no known competing financial interests or personal relationships that could have appeared to influence the work reported in this paper.
