# Supplementary material for: Amino acid sequence homology between thyroid autoantigens and central nervous system proteins: Implications for the steroid-responsive encephalopathy associated with autoimmune thyroiditis
Source: J Clin Transl Endocrinol. 2021 Nov 6;26:100274. doi: 10.1016/j.jcte.2021.100274 (PMC8609095; doi:10.1016/j.jcte.2021.100274)
Supplement: Supplementary data 1 [file mmc1.doc]

**Supplementary Table 1.** Expression in central nervous system and thyroid, as resulting from a search in the Expression Atlas (https://www.ebi.ac.uk/gxa/home) [31], of the proteins that we found share local homology with thyrotropin receptor (TSH-R). Central nervous system areas whose involvement in Hashimoto’s encephalopathy is reported in literature are highlighted in gray.

|  | Expressed in | | | | | | | | | | | | | | | | | | | | | | | | | | | | | | | | | | | | | |
| --- | --- | --- | --- | --- | --- | --- | --- | --- | --- | --- | --- | --- | --- | --- | --- | --- | --- | --- | --- | --- | --- | --- | --- | --- | --- | --- | --- | --- | --- | --- | --- | --- | --- | --- | --- | --- | --- | --- |
| Protein [Entrez Protein GI accession number] | amygdala | brain | brain meninx | Brodmann (1909) area 24 | Brodmann (1909) area 9 | caudate nucleus | cerebellar hemisphere | cerebellum | cerebral cortex | diencephalon | dorsal thalamus | dorsolateral prefrontal cortex | dura mater | entorhinal cortex | frontal cortex | frontal lobe | globus pallidus | hippocampus | hypothalamus | locus ceruleus | medulla oblongata | middle frontal gyrus | middle temporal gyrus | nucleus accumbens | occipital cortex | occipital lobe | parietal lobe | pineal body | pituitary gland | prefrontal cortex | primary visual cortex | putamen | substantia nigra | telencephalic ventricle | temporal lobe | thalamus | visual cortex | thyroid |
| Leucine-rich repeat-containing G-protein coupled receptor 4 (LGR4) [157694513] | √ | √ |  | √ | √ | √ | √ | √ | √ | √ |  |  | √ |  | √ | √ |  | √ | √ | √ |  |  |  | √ |  | √ |  |  | √ | √ |  | √ | √ |  | √ |  |  | √ |
| Leucine-rich repeat-containing G-protein coupled receptor 5 (LGR5) [4504379] | √ | √ | √ | √ | √ | √ | √ | √ | √ | √ | √ |  | √ |  | √ | √ | √ | √ | √ | √ | √ | √ | √ | √ | √ | √ | √ | √ | √ | √ |  | √ | √ |  | √ |  |  | √ |
| Relaxin receptor 2 / Leucine-rich repeat-containing G-protein coupled receptor 8 (LGR8) [18677729] |  |  |  |  |  |  |  |  |  |  |  |  |  |  |  |  |  |  |  |  |  |  |  |  |  |  |  |  |  |  |  | √ |  |  |  |  |  |  |
| Relaxin receptor 1 / Leucine-rich repeat-containing G protein-coupled receptor 7 (LGR7) [359279868] | √ | √ |  | √ | √ |  |  |  | √ |  |  |  |  |  | √ | √ |  | √ |  |  |  | √ | √ | √ |  | √ |  |  |  | √ |  |  |  |  | √ |  |  | √ |
| Chondroadherin [153251229] | √ | √ | √ | √ | √ | √ | √ | √ | √ | √ | √ |  | √ | √ | √ | √ | √ | √ | √ | √ | √ | √ | √ | √ | √ | √ | √ |  | √ | √ |  | √ | √ | √ | √ | √ | √ | √ |
| Leucine-rich repeat and immunoglobulin-like domain-containing nogo receptor-interacting protein 2 precursor (LINGO2) [22749183] | √ | √ |  | √ | √ | √ | √ | √ | √ |  |  | √ |  | √ | √ | √ |  | √ | √ |  |  | √ |  | √ |  |  | √ |  | √ | √ | √ |  | √ |  | √ | √ | √ | √ |
| Somatostatin receptor type 2 [4557859] | √ | √ | √ | √ | √ | √ | √ | √ | √ | √ |  |  |  | √ | √ | √ |  | √ | √ | √ | √ | √ | √ | √ |  | √ | √ | √ | √ | √ |  | √ | √ |  | √ | √ | √ | √ |
| Neuropeptide Y receptor type 1 [4505445] | √ | √ |  | √ | √ | √ | √ | √ | √ | √ |  |  |  |  | √ | √ |  | √ | √ |  |  | √ | √ | √ | √ |  | √ |  | √ | √ |  | √ | √ |  | √ |  |  | √ |
| Apelin receptor [4885057] | √ | √ | √ | √ | √ | √ | √ | √ | √ | √ | √ |  | √ | √ | √ | √ | √ | √ | √ | √ | √ | √ | √ | √ | √ | √ | √ | √ | √ | √ |  | √ | √ |  | √ | √ | √ | √ |
| Neuromedin-K receptor / Neurokinin B receptor / Tachykinin receptor 3 [7669548] | √ | √ |  |  |  |  |  |  | √ |  |  |  |  |  |  | √ |  |  | √ | √ |  | √ |  |  |  |  |  |  |  |  |  | √ | √ |  |  |  |  |  |
| Free fatty acid receptor 3 [4885329] |  |  |  |  |  |  |  |  |  |  |  |  |  |  |  |  |  |  |  |  |  |  |  |  |  |  |  |  |  |  |  |  |  |  |  |  |  |  |
| Melanopsin / Opsin-4 [15150803] | √ | √ |  | √ | √ | √ |  |  | √ |  |  |  |  |  | √ | √ | √ | √ |  |  |  | √ | √ | √ |  |  |  |  | √ | √ |  | √ |  |  | √ |  |  | √ |
| G-protein coupled estrogen receptor 1 / Membrane estrogen receptor [4504091] | √ | √ |  | √ | √ | √ | √ | √ | √ |  |  |  | √ |  | √ | √ |  | √ | √ |  | √ |  |  | √ | √ |  |  |  | √ | √ |  | √ | √ |  | √ |  |  | √ |
| Alpha-1A adrenergic receptor [111118992] | √ | √ |  | √ | √ | √ | √ | √ | √ |  |  |  |  | √ | √ | √ |  | √ | √ | √ |  | √ |  | √ |  |  | √ | √ |  | √ |  | √ | √ |  | √ | √ | √ | √ |
| Vasopressin V1a receptor [4502331] |  | √ |  |  |  |  |  |  | √ |  |  |  |  |  | √ | √ |  |  |  |  |  |  |  |  |  |  |  |  | √ | √ |  |  |  |  |  |  |  | √ |
| Probable G-protein coupled receptor 34 [4885319] | √ | √ | √ | √ | √ | √ | √ | √ | √ | √ | √ |  | √ |  | √ | √ | √ | √ | √ | √ | √ | √ | √ | √ | √ | √ | √ | √ | √ | √ |  | √ | √ |  | √ |  |  | √ |
| G-protein coupled receptor 26 [23592220] | √ | √ |  | √ | √ |  |  |  | √ |  |  |  |  |  | √ | √ |  | √ | √ |  |  |  |  |  |  |  |  |  |  | √ |  |  | √ |  | √ |  |  |  |
| Orexin 2 receptor [1285033761] |  |  |  |  |  |  |  | √ | √ |  |  |  |  |  | √ | √ |  |  | √ |  |  |  |  |  |  |  |  |  |  | √ |  |  |  |  | √ |  |  |  |
| Oxytocin receptor [32307152] | √ | √ |  | √ | √ | √ | √ | √ | √ |  |  |  |  |  | √ | √ |  | √ | √ |  |  |  |  | √ |  |  |  |  | √ | √ |  | √ | √ |  | √ |  |  | √ |
| Orexin receptor type 1 [222080095] | √ |  |  |  |  |  |  |  | √ |  |  |  |  |  | √ | √ |  |  | √ | √ |  | √ |  |  | √ |  |  |  | √ | √ |  |  |  |  | √ |  |  | √ |
| Galanin receptor type 2 [4503905] |  |  |  |  |  |  |  | √ |  |  |  |  |  |  |  | √ |  |  | √ |  |  |  |  |  |  |  |  |  |  |  |  |  |  |  |  |  |  |  |
| GPER protein [52350636] | √ | √ |  | √ | √ | √ | √ | √ | √ |  |  |  | √ |  | √ | √ |  | √ | √ |  | √ |  |  | √ | √ |  |  |  | √ | √ |  | √ | √ |  | √ |  |  | √ |
| N/OFQ opioid receptor [385252102] | √ | √ |  | √ | √ | √ | √ | √ | √ |  |  |  |  |  | √ | √ |  | √ | √ |  |  |  |  | √ |  |  |  |  | √ | √ |  | √ | √ |  | √ |  |  | √ |
| Type 2 angiotensin II receptor [23238240] |  |  |  |  |  |  |  | √ |  |  |  |  |  |  |  |  |  |  |  |  |  |  |  |  |  |  |  |  |  |  |  |  |  |  |  |  |  |  |
| Alpha-1B adrenergic receptor [4501959] | √ | √ |  | √ | √ | √ | √ | √ | √ |  |  |  |  | √ | √ | √ |  | √ | √ |  |  | √ |  | √ |  |  | √ |  | √ | √ |  | √ | √ |  | √ | √ | √ | √ |
| Mu opioid receptor [119568090] | √ |  |  |  |  | √ | √ | √ | √ | √ | √ |  |  | √ | √ | √ |  |  |  | √ |  | √ | √ |  |  |  | √ |  |  | √ |  |  |  |  | √ | √ |  |  |
| Melanin-concentrating hormone receptor 1 [397487122] | √ | √ | √ | √ | √ |  | √ | √ | √ |  |  |  |  |  | √ | √ |  | √ | √ |  |  | √ | √ | √ | √ | √ | √ | √ | √ | √ |  |  | √ |  | √ |  |  |  |
| Bombesin receptor subtype-3 [4502455] |  | √ |  |  |  |  |  |  |  |  |  |  |  |  |  |  |  |  | √ |  |  |  |  | √ |  |  |  |  |  |  |  | √ |  |  |  |  |  |  |
| Neuropeptide Y receptor type 5 [5453796] | √ | √ | √ | √ | √ | √ |  |  | √ | √ | √ |  | √ |  | √ | √ | √ | √ | √ | √ | √ | √ | √ | √ |  | √ | √ |  |  | √ |  | √ |  |  | √ |  |  | √ |
| C3a anaphylatoxin chemotactic receptor [4757888] | √ | √ | √ | √ | √ | √ | √ | √ | √ | √ | √ |  | √ |  | √ | √ | √ | √ | √ | √ | √ | √ | √ | √ | √ | √ | √ | √ | √ | √ |  | √ | √ |  | √ |  |  | √ |
| Substance-P receptor / Tachykinin receptor 1 [4507343] | √ | √ |  | √ | √ | √ |  |  | √ |  |  |  |  | √ | √ | √ |  | √ | √ |  |  | √ |  | √ |  |  | √ |  |  | √ |  | √ |  |  | √ | √ | √ | √ |
| Proteinase-activated receptor 2 [34577052] | √ | √ | √ | √ |  | √ |  |  | √ |  | √ |  | √ |  | √ |  |  | √ | √ |  | √ |  |  | √ |  | √ | √ | √ | √ | √ |  | √ | √ |  |  |  |  | √ |
| Trace amine-associated receptor 6 (TaR-6) [28173558] |  |  |  |  |  |  |  |  |  |  |  |  |  |  |  |  |  |  |  |  |  |  |  |  |  |  |  |  |  |  |  |  |  |  |  |  |  |  |
| Urotensin-2 receptor / G-protein coupled receptor 14 [9506745] |  |  |  |  | √ |  |  | √ | √ |  |  |  |  |  | √ | √ |  |  |  |  |  |  |  |  |  |  |  |  |  | √ |  |  |  |  | √ |  |  | √ |
| Nociceptin receptor [974065167] | √ | √ |  | √ | √ | √ | √ | √ | √ |  |  |  |  |  | √ | √ |  | √ | √ |  |  |  |  | √ |  |  |  |  | √ | √ |  | √ | √ |  | √ |  |  | √ |
| G-protein coupled receptor 24 [56554976] | √ | √ | √ | √ | √ |  | √ | √ | √ |  |  |  |  |  | √ | √ |  | √ | √ |  |  | √ | √ | √ | √ | √ | √ | √ | √ | √ |  |  | √ |  | √ |  |  |  |
| C-C chemokine receptor type 7 / Epstein-Barr virus-induced G-protein coupled receptor 1 / MIP-3 beta receptor [4502641] |  |  |  |  |  |  |  |  | √ |  |  |  | √ |  |  | √ |  |  |  |  |  |  |  |  |  |  |  |  | √ |  |  |  |  |  |  |  |  | √ |
| Olfactory receptor 2A14 [48717236] |  |  |  |  |  |  |  |  |  |  |  |  |  |  |  |  |  |  |  |  |  |  |  |  |  |  |  |  |  |  |  |  |  |  |  |  |  |  |
| Vasopressin V2 receptor [4557345] |  |  |  |  |  |  | √ | √ |  |  |  |  |  |  |  | √ |  |  |  |  |  |  |  |  |  |  |  |  |  |  |  |  |  |  | √ |  |  | √ |
| Neuropeptide S receptor [46395496] |  | √ |  |  |  |  |  |  |  |  |  |  |  |  |  |  |  |  |  |  |  |  |  |  |  |  |  |  |  |  |  |  |  |  | √ |  |  |  |
| Trace amine-associated receptor 8 (TaR-8) [16751917] |  |  |  |  |  |  |  |  |  |  |  |  |  |  |  |  |  |  |  |  |  |  |  |  |  |  |  |  |  |  |  |  |  |  |  |  |  |  |
| Neuropeptides B/W receptor type 2 [30581164] |  | √ | √ |  | √ |  |  |  | √ |  |  |  |  |  | √ | √ |  |  |  |  |  | √ | √ |  | √ | √ | √ |  |  | √ |  |  |  |  | √ |  |  |  |
| Olfactory receptor 2J3 [185134902] |  |  |  |  |  |  |  |  |  |  |  |  |  |  |  |  |  |  |  |  |  |  |  |  |  |  |  |  |  |  |  |  |  |  |  |  |  |  |
| G protein-coupled receptor [953233] |  |  |  |  |  |  |  |  |  |  |  |  |  |  | √ | √ |  |  |  |  |  |  |  |  |  |  |  |  | √ | √ |  |  |  |  | √ |  |  |  |
| Oxoglutarate (alpha-ketoglutarate) receptor 1 [52426789] |  | √ |  | √ | √ |  |  |  | √ |  |  |  |  |  | √ | √ |  |  |  |  |  |  |  |  |  |  |  |  | √ | √ |  |  |  |  | √ |  |  | √ |
| 5-hydroxytryptamine receptor 7 (5-HT7) [10880129] |  | √ |  | √ | √ |  |  | √ | √ |  |  |  |  |  | √ | √ |  | √ | √ |  |  |  |  |  |  |  |  |  |  | √ |  |  |  | √ | √ |  |  | √ |
