# Supplementary material for: Amino acid sequence homology between thyroid autoantigens and central nervous system proteins: Implications for the steroid-responsive encephalopathy associated with autoimmune thyroiditis
Source: J Clin Transl Endocrinol. 2021 Nov 6;26:100274. doi: 10.1016/j.jcte.2021.100274 (PMC8609095; doi:10.1016/j.jcte.2021.100274)
Supplement: Supplementary data 2 [file mmc2.doc]

**Supplementary Table 2.** Expression in central nervous system and thyroid, as resulting from a search in the Expression Atlas (https://www.ebi.ac.uk/gxa/home) [31], of the proteins that we found share local homology with thyroglobulin (Tg). Central nervous system areas whose involvement in Hashimoto’s encephalopathy is reported in literature are highlighted in gray.

|  | Expressed in | | | | | | | | | | | | | | | | | | | | | | | | | | | | | | | | | | | | | | | | | | | |
| --- | --- | --- | --- | --- | --- | --- | --- | --- | --- | --- | --- | --- | --- | --- | --- | --- | --- | --- | --- | --- | --- | --- | --- | --- | --- | --- | --- | --- | --- | --- | --- | --- | --- | --- | --- | --- | --- | --- | --- | --- | --- | --- | --- | --- |
| **Protein [Entrez Protein GI accession number]** | amygdala | basal ganglion | brain | brain meninx | Brodmann (1909) area 24 | Brodmann (1909) area 9 | caudate nucleus | cerebellar hemisphere | cerebellum | cerebral cortex | choroid plexus | diencephalon | dorsal thalamus | dorsolateral prefrontal cortex | dura mater | entorhinal cortex | forebrain fragment | frontal cortex | frontal lobe | globus pallidus | hippocampus | hypothalamus | locus ceruleus | medulla oblongata | midbrain | middle frontal gyrus | middle temporal gyrus | nucleus accumbens | occipital cortex | occipital lobe | parietal lobe | pineal body | pituitary gland | prefrontal cortex | primary visual cortex | putamen | striatum | substantia nigra | telencephalic ventricle | temporal cortex | temporal lobe | thalamus | visual cortex | thyroid |
| Nidogen-1 / Entactin [115298674] | √ |  | √ | √ | √ | √ | √ | √ | √ | √ |  | √ | √ | √ | √ | √ |  | √ | √ | √ | √ | √ | √ | √ |  | √ | √ | √ | √ | √ | √ | √ | √ | √ | √ | √ | √ | √ |  | √ | √ | √ | √ | √ |
| Testican-1 / Protein SPOCK [4759164] | √ |  | √ |  | √ | √ | √ | √ | √ | √ |  |  |  |  |  | √ |  | √ | √ |  | √ | √ |  |  |  | √ |  | √ |  |  | √ | √ | √ | √ | √ | √ | √ | √ |  |  | √ | √ | √ | √ |
| Testican-2 / SPARC/osteonectin, CWCV, and Kazal-like domains proteoglycan 2 (SPOCK2) [7662036] | √ |  | √ | √ | √ | √ | √ | √ | √ | √ |  | √ | √ |  |  | √ |  | √ | √ | √ | √ | √ | √ | √ |  | √ | √ | √ | √ | √ | √ |  | √ | √ |  | √ |  | √ |  |  | √ | √ | √ | √ |
| SPARC-related modular calcium-binding protein 1 / Secreted modular calcium-binding protein 1 (SMOC-1) [11545873] | √ |  | √ | √ | √ | √ | √ | √ | √ | √ |  | √ | √ |  | √ | √ |  | √ | √ | √ | √ | √ | √ | √ |  | √ | √ | √ | √ | √ | √ | √ | √ | √ |  | √ |  | √ |  |  | √ | √ | √ | √ |
| Testican-3 [3581970] | √ |  | √ | √ | √ | √ | √ | √ | √ | √ |  | √ | √ |  |  | √ |  | √ | √ | √ | √ | √ | √ | √ |  | √ | √ | √ | √ | √ | √ | √ | √ | √ |  | √ |  | √ |  |  | √ | √ | √ | √ |
| SPARC-related modular calcium-binding protein 2 / Secreted modular calcium-binding protein 2 (SMOC-2) [262050673] | √ |  | √ | √ | √ | √ | √ | √ | √ | √ |  | √ | √ |  | √ |  |  | √ | √ | √ | √ | √ | √ | √ |  | √ | √ | √ | √ | √ |  | √ | √ | √ |  | √ |  | √ |  |  | √ |  |  | √ |
| Insulin-like growth factor-binding protein 5 [10834982] | √ |  | √ |  | √ | √ | √ | √ | √ | √ |  |  |  |  | √ | √ |  | √ | √ |  | √ | √ |  |  |  | √ |  | √ |  |  | √ |  | √ | √ |  | √ |  | √ |  |  | √ | √ | √ | √ |
| Signal peptide, CUB and EGF-like domain-containing protein 1 [120587029] |  |  | √ |  |  |  |  |  |  |  |  |  |  |  |  |  |  | √ | √ |  |  | √ |  |  |  |  |  |  |  |  |  |  | √ | √ |  |  |  | √ |  |  | √ |  |  | √ |
| Ephrin type-B receptor 2 [822606583] | √ |  | √ | √ | √ | √ | √ | √ | √ | √ |  | √ | √ | √ | √ | √ |  | √ | √ | √ | √ | √ | √ | √ |  | √ | √ | √ | √ | √ | √ | √ | √ | √ | √ | √ | √ | √ |  | √ | √ | √ | √ | √ |
| Ephrin type-B receptor 6 [294862532] | √ |  | √ | √ | √ | √ | √ | √ | √ | √ |  | √ | √ | √ | √ |  |  | √ | √ | √ | √ | √ | √ | √ |  | √ | √ | √ | √ | √ | √ | √ | √ | √ | √ | √ | √ | √ |  |  | √ |  | √ | √ |
| Ephrin type-A receptor 7 [568599847] | √ |  | √ | √ | √ | √ | √ | √ | √ | √ |  | √ | √ | √ | √ | √ |  | √ | √ | √ | √ | √ | √ | √ |  | √ | √ | √ | √ | √ | √ | √ | √ | √ |  | √ |  | √ |  |  | √ | √ | √ | √ |
| Acetylcholinesterase (Yt blood group) [219518823] | √ |  | √ |  | √ | √ | √ | √ | √ | √ |  |  |  | √ |  | √ |  | √ | √ |  | √ | √ |  |  |  | √ |  | √ |  |  | √ |  | √ | √ |  | √ | √ | √ | √ |  | √ | √ | √ | √ |
| Butyrylcholinesterase [1073548962] | √ |  | √ | √ | √ | √ | √ | √ | √ | √ |  | √ | √ |  | √ | √ |  | √ | √ | √ | √ | √ | √ | √ |  | √ | √ | √ | √ | √ | √ | √ | √ | √ |  | √ |  | √ |  |  | √ | √ | √ | √ |
| Neuroligin-3 [262359974] | √ |  | √ | √ | √ | √ | √ | √ | √ | √ |  | √ | √ | √ | √ | √ |  | √ | √ | √ | √ | √ | √ | √ |  | √ | √ | √ | √ | √ | √ | √ | √ | √ | √ | √ | √ | √ | √ | √ | √ | √ | √ | √ |
| Neuroligin-4, X-linked [24308209] | √ |  | √ | √ | √ | √ | √ | √ | √ | √ |  | √ | √ | √ | √ | √ |  | √ | √ | √ | √ | √ | √ | √ |  | √ | √ | √ | √ | √ | √ | √ | √ | √ | √ | √ |  | √ |  |  | √ | √ | √ | √ |
| Neuroligin-4, Y-linked [256222771] | √ |  | √ | √ | √ | √ | √ | √ | √ | √ |  | √ |  |  |  | √ |  | √ | √ |  | √ | √ |  |  |  | √ |  | √ |  | √ | √ |  | √ | √ |  | √ |  | √ |  |  | √ | √ | √ | √ |
| Neuroligin-1 [1478051093] | √ |  | √ | √ | √ | √ | √ | √ | √ | √ |  | √ |  |  |  | √ |  | √ | √ | √ | √ | √ | √ | √ |  | √ | √ | √ | √ | √ | √ |  | √ | √ |  | √ | √ | √ |  |  | √ | √ | √ | √ |
| Carboxylesterase 3 (CES3) [297747275] | √ |  | √ |  | √ | √ | √ | √ | √ | √ |  |  |  |  |  |  |  | √ | √ |  | √ | √ |  |  |  |  |  | √ | √ |  |  | √ | √ | √ |  | √ |  | √ | √ |  | √ |  |  | √ |
| Cocaine esterase [1463570077] | √ |  | √ | √ | √ | √ | √ | √ | √ | √ |  | √ | √ |  | √ | √ |  | √ | √ | √ | √ | √ | √ | √ |  | √ | √ | √ | √ | √ | √ | √ | √ | √ |  | √ |  | √ | √ |  | √ | √ | √ | √ |
| Carboxylesterase 5A [298231153] |  |  |  |  |  |  |  |  |  | √ |  |  |  |  |  |  |  |  | √ |  |  |  |  |  |  |  |  |  |  |  |  |  |  |  |  |  |  |  |  |  | √ |  |  |  |
| Neuroligin-2 [30840978] | √ |  | √ |  | √ | √ | √ | √ | √ | √ |  |  |  |  |  |  |  | √ | √ |  | √ | √ |  |  |  | √ | √ | √ |  |  |  |  | √ | √ |  | √ |  | √ |  |  | √ |  |  | √ |
| Brain carboxylesterase hBr3 [6009628] |  |  | √ |  |  |  |  |  |  |  |  |  |  |  |  |  |  |  |  |  |  |  |  |  |  |  |  |  |  |  |  |  |  |  |  |  |  |  |  |  |  |  |  |  |
| Liver carboxylesterase 1 / Acyl-coenzyme A:cholesterol acyltransferase / Brain carboxylesterase hBr1 / Cocaine carboxylesterase / Egasyn / Methylumbelliferyl-acetate deacetylase 1 / Monocyte/macrophage serine esterase / Retinyl ester hydrolase / Serine esterase 1 / Triacylglycerol hydrolase [68508965] |  | √ | √ |  |  |  | √ |  | √ | √ | √ | √ |  |  |  |  | √ | √ | √ |  | √ |  |  |  | √ |  |  |  |  |  |  | √ | √ | √ |  |  |  |  | √ |  | √ |  |  | √ |
| KIAA1480 protein, partial [7959221] | √ |  | √ | √ | √ | √ | √ | √ | √ | √ |  | √ | √ | √ | √ | √ |  | √ | √ | √ | √ | √ | √ | √ |  | √ | √ | √ | √ | √ | √ | √ | √ | √ | √ | √ | √ | √ | √ | √ | √ | √ | √ | √ |
| Carboxylesterase 4A [1419235141] | √ |  | √ | √ | √ | √ | √ | √ | √ | √ |  |  | √ |  |  |  |  | √ | √ | √ | √ | √ | √ |  |  | √ | √ | √ | √ |  | √ | √ | √ | √ |  | √ |  | √ |  |  | √ |  |  | √ |
| Carboxylesterase 8 (CES8) [40555853] | √ |  | √ | √ | √ | √ | √ | √ | √ | √ |  |  | √ |  |  |  |  | √ | √ | √ | √ | √ | √ |  |  | √ | √ | √ | √ |  | √ | √ | √ | √ |  | √ |  | √ |  |  | √ |  |  | √ |
| KIAA1366 protein, partial [7243113] | √ |  | √ |  | √ | √ | √ | √ | √ | √ |  |  |  |  |  |  |  | √ | √ |  | √ | √ |  |  |  | √ | √ | √ |  |  |  |  | √ | √ |  | √ |  | √ |  |  | √ |  |  | √ |
