# Supplementary material for: Amino acid sequence homology between thyroid autoantigens and central nervous system proteins: Implications for the steroid-responsive encephalopathy associated with autoimmune thyroiditis
Source: J Clin Transl Endocrinol. 2021 Nov 6;26:100274. doi: 10.1016/j.jcte.2021.100274 (PMC8609095; doi:10.1016/j.jcte.2021.100274)
Supplement: Supplementary data 3 [file mmc3.doc]

**Supplementary Table 3.** Expression in central nervous system and thyroid, as resulting from a search in the Expression Atlas (https://www.ebi.ac.uk/gxa/home) [31], of the proteins that we found share local homology with thyroperoxidase (TPO). Central nervous system areas whose involvement in Hashimoto’s encephalopathy is reported in literature are highlighted in gray.

|  | Expressed in | | | | | | | | | | | | | | | | | | | | | | | | | | | | | | | | | | | | | | | |
| --- | --- | --- | --- | --- | --- | --- | --- | --- | --- | --- | --- | --- | --- | --- | --- | --- | --- | --- | --- | --- | --- | --- | --- | --- | --- | --- | --- | --- | --- | --- | --- | --- | --- | --- | --- | --- | --- | --- | --- | --- |
| **Protein [Entrez Protein GI accession number]** | amygdala | brain | brain meninx | Brodmann (1909) area 24 | Brodmann (1909) area 9 | caudate nucleus | cerebellar hemisphere | cerebellum | cerebral cortex | diencephalon | dorsal thalamus | dorsolateral prefrontal cortex | dura mater | entorhinal cortex | frontal cortex | frontal lobe | globus pallidus | hippocampus | hypothalamus | locus ceruleus | medulla oblongata | middle frontal gyrus | middle temporal gyrus | nucleus accumbens | occipital cortex | occipital lobe | parietal lobe | pineal body | pituitary gland | prefrontal cortex | primary visual cortex | putamen | striatum | substantia nigra | telencephalic ventricle | temporal cortex | temporal lobe | thalamus | visual cortex | thyroid |
| Peroxidasin homolog / Melanoma-associated antigen MG50 / Vascular peroxidase 1 [109150416] | √ | √ | √ | √ | √ | √ | √ | √ | √ | √ | √ |  | √ | √ | √ | √ | √ | √ | √ | √ | √ | √ | √ | √ | √ | √ | √ | √ | √ | √ |  | √ |  | √ |  |  | √ | √ | √ | √ |
| Peroxidasin-like protein [633365073] |  | √ |  |  |  |  |  |  | √ |  |  |  |  |  | √ | √ |  |  |  |  |  |  |  |  |  |  |  |  | √ | √ |  |  |  |  |  |  | √ |  |  | √ |
| Prostaglandin G/H synthase 2 / Cyclooxygenase-2 [4506265] | √ | √ | √ | √ | √ | √ |  | √ | √ | √ | √ |  | √ |  | √ | √ | √ | √ | √ | √ | √ | √ | √ | √ | √ | √ | √ | √ | √ | √ |  | √ |  | √ | √ |  | √ |  |  | √ |
| Prostaglandin G/H synthase 1 / Cyclooxygenase-1 [18104967] | √ | √ | √ | √ | √ | √ | √ | √ | √ | √ | √ |  | √ | √ | √ | √ | √ | √ | √ | √ | √ | √ | √ | √ | √ | √ | √ | √ | √ | √ | √ | √ |  | √ | √ |  | √ | √ | √ | √ |
| Fibrillin-1 / Asprosin / Epididymis secretory sperm binding protein [311033452] | √ | √ |  | √ | √ | √ | √ | √ | √ | √ | √ |  | √ | √ | √ | √ | √ | √ | √ | √ |  | √ |  | √ |  |  | √ |  | √ | √ |  | √ |  | √ |  |  | √ | √ | √ | √ |
| Adhesion G protein-coupled receptor E2 / EGF-like module receptor 2 / CD312 [23397681] |  | √ |  |  |  |  |  |  | √ |  |  |  |  |  |  | √ |  |  | √ |  |  |  |  |  |  |  |  |  | √ |  |  |  |  | √ |  |  | √ |  |  | √ |
| Protocadherin Fat 4 [165932370] | √ | √ |  | √ | √ | √ |  | √ | √ |  |  |  |  |  | √ | √ |  | √ | √ |  |  |  |  | √ |  |  |  |  | √ | √ |  | √ |  | √ |  |  | √ |  |  | √ |
| Low-density lipoprotein receptor-related protein 4 (LRP-4) [157384998] | √ | √ |  | √ | √ | √ | √ | √ | √ |  |  |  |  | √ | √ | √ |  | √ | √ |  |  | √ |  | √ |  |  | √ |  | √ | √ |  | √ |  | √ | √ |  | √ | √ | √ | √ |
| Latent-transforming growth factor beta-binding protein 4 (LTBP4) [110347431] | √ | √ |  | √ | √ | √ | √ | √ | √ | √ | √ |  | √ |  | √ | √ |  | √ | √ |  | √ |  |  | √ |  |  |  | √ | √ | √ |  | √ |  | √ | √ |  | √ |  |  | √ |
| Fibrillin-3 [56237021] | √ | √ |  | √ | √ |  |  |  | √ |  |  |  |  |  | √ | √ |  | √ | √ |  |  |  |  |  |  |  |  |  | √ | √ |  |  |  | √ |  |  | √ |  |  | √ |
| Latent-transforming growth factor beta-binding protein 1 (LTBP-1) [290457687] | √ | √ | √ | √ | √ | √ | √ | √ | √ | √ | √ |  | √ | √ | √ | √ | √ | √ | √ | √ | √ | √ |  | √ |  |  | √ | √ | √ | √ |  | √ |  | √ |  |  | √ | √ | √ | √ |
| Seizure related 6-like protein 2 [608785583] | √ | √ | √ | √ | √ | √ | √ | √ | √ | √ | √ | √ |  | √ | √ | √ | √ | √ | √ | √ | √ | √ | √ | √ | √ | √ | √ | √ | √ | √ | √ | √ | √ | √ | √ | √ | √ | √ | √ | √ |
| CUB and sushi domain-containing protein 1 [259013213] | √ | √ |  | √ | √ | √ | √ | √ | √ |  |  |  |  | √ | √ | √ |  | √ | √ |  |  | √ | √ | √ |  | √ | √ |  |  | √ |  | √ |  | √ |  |  | √ | √ | √ | √ |
| C-type lectin domain family 14 member A / Epidermal growth factor receptor 5 (EGFR-5) [28269707] | √ | √ |  | √ | √ | √ | √ | √ | √ | √ |  |  | √ | √ | √ | √ |  | √ | √ |  | √ | √ |  | √ |  |  | √ |  | √ | √ |  | √ |  | √ | √ |  | √ | √ | √ | √ |
| fibrillin 1 variant, partial [62087260] | √ | √ |  | √ | √ | √ | √ | √ | √ | √ | √ |  | √ | √ | √ | √ | √ | √ | √ | √ |  | √ |  | √ |  |  | √ |  | √ | √ |  | √ |  | √ |  |  | √ | √ | √ | √ |
| Multiple epidermal growth factor-like domains protein 6 [110347457] | √ | √ |  | √ | √ | √ | √ | √ | √ |  |  |  |  |  | √ | √ |  | √ | √ |  |  |  |  | √ |  |  |  |  | √ | √ |  | √ |  | √ |  |  | √ |  |  | √ |
| Seizure 6-like protein / KIAA0927 protein [296179442] | √ | √ | √ | √ | √ | √ | √ | √ | √ | √ | √ | √ | √ | √ | √ | √ | √ | √ | √ | √ | √ | √ | √ | √ | √ | √ | √ | √ | √ | √ | √ | √ |  | √ |  | √ | √ | √ | √ | √ |
| Cadherin EGF LAG seven-pass G-type receptor 2 / Cadherin family member 10 / Flamingo homolog 3 [13325064] | √ | √ | √ | √ | √ | √ | √ | √ | √ | √ | √ | √ |  | √ | √ | √ | √ | √ | √ | √ | √ | √ | √ | √ | √ | √ | √ |  | √ | √ |  | √ |  | √ |  |  | √ | √ | √ | √ |
| Low-density lipoprotein receptor-related protein 2 (LRP-2) [126012573] | √ | √ |  | √ | √ | √ | √ | √ | √ | √ | √ |  |  | √ | √ | √ | √ | √ | √ | √ | √ | √ | √ |  | √ | √ | √ |  | √ | √ | √ | √ | √ | √ |  |  | √ | √ | √ | √ |
| EGF-containing fibulin-like extracellular matrix protein 2 [14714634] | √ | √ | √ | √ | √ | √ | √ | √ | √ | √ |  |  | √ |  | √ | √ |  | √ | √ |  |  |  |  | √ |  |  |  |  | √ | √ |  | √ |  | √ |  |  | √ |  |  | √ |
| Nephronectin / Preosteoblast EGF-like repeat protein with MAM domain / EGFL6-like [75709198] | √ | √ |  | √ | √ | √ |  | √ | √ |  |  |  | √ | √ | √ | √ |  | √ | √ |  |  | √ |  | √ |  |  | √ |  | √ | √ |  | √ |  | √ |  |  | √ | √ | √ | √ |
| Complement component C1q receptor / CD93 [88758613] | √ | √ |  | √ | √ | √ | √ | √ | √ |  |  |  | √ |  | √ | √ |  | √ | √ |  | √ | √ |  | √ |  |  | √ |  | √ | √ |  | √ |  | √ |  |  | √ | √ | √ | √ |
| Fibulin 5 [19743803] | √ | √ | √ | √ | √ | √ | √ | √ | √ |  |  |  | √ | √ | √ | √ |  | √ | √ |  | √ | √ |  | √ |  |  | √ | √ | √ | √ |  | √ |  | √ |  |  | √ | √ | √ | √ |
| Tolloid-like protein 1 [22547221] |  | √ |  |  | √ |  | √ | √ | √ |  |  |  |  |  | √ |  |  | √ | √ |  |  |  |  |  |  |  |  |  | √ | √ |  |  |  |  |  |  | √ |  |  | √ |
| EGF-containing fibulin-like extracellular matrix protein 1 [86788015] | √ | √ | √ | √ | √ | √ | √ | √ | √ | √ | √ | √ | √ | √ | √ | √ | √ | √ | √ | √ | √ | √ | √ | √ | √ | √ | √ | √ | √ | √ | √ | √ | √ | √ | √ | √ | √ | √ | √ | √ |
| Signal peptide, CUB and EGF-like domain-containing protein 1 [120587029] |  | √ |  |  |  |  |  |  |  |  |  |  |  |  | √ | √ |  |  | √ |  |  |  |  |  |  |  |  |  | √ | √ |  |  |  | √ |  |  | √ |  |  | √ |
| Latent-transforming growth factor beta-binding protein 1 (LTBP1) [219518146] | √ | √ | √ | √ | √ | √ | √ | √ | √ | √ | √ |  | √ | √ | √ | √ | √ | √ | √ | √ | √ | √ |  | √ |  |  | √ | √ | √ | √ |  | √ |  | √ |  |  | √ | √ | √ | √ |
| KIAA1237 protein, partial [34327974] | √ | √ |  | √ | √ | √ | √ | √ | √ | √ | √ |  | √ | √ | √ | √ | √ | √ | √ | √ | √ | √ |  | √ |  |  | √ | √ | √ | √ |  | √ |  | √ |  |  | √ | √ | √ | √ |
| Vitamin K-dependent protein S [192447438] | √ | √ | √ | √ | √ | √ | √ | √ | √ |  |  |  | √ | √ | √ | √ |  | √ | √ |  | √ | √ |  | √ |  |  | √ |  | √ | √ |  | √ |  | √ |  |  | √ | √ | √ | √ |
| Protein HEG homolog 1 [153792110] | √ | √ |  | √ | √ | √ | √ | √ | √ | √ | √ |  | √ | √ | √ | √ | √ | √ | √ | √ | √ | √ |  | √ |  |  | √ | √ | √ | √ |  | √ |  | √ |  |  | √ | √ | √ | √ |
| Low-density lipoprotein receptor-related protein 1B (LRP-1B) [93102379] | √ | √ | √ | √ | √ | √ | √ | √ | √ | √ | √ |  | √ | √ | √ | √ | √ | √ | √ | √ | √ | √ | √ | √ | √ | √ | √ |  | √ | √ |  | √ | √ | √ |  |  | √ | √ | √ | √ |
| P-selectin (CD62P) / Granule membrane protein 140 / Leukocyte-endothelial cell adhesion molecule 3 / Platelet activation dependent granule-external membrane protein [215274139] |  | √ |  |  |  |  |  |  |  |  |  |  | √ |  |  |  |  |  |  |  |  |  |  |  |  |  |  |  | √ |  |  |  |  |  |  |  |  |  |  | √ |
| Fibulin-1 (FIBL-1) [215274249] | √ | √ |  | √ | √ | √ | √ | √ | √ |  |  |  | √ | √ | √ | √ |  | √ | √ |  |  | √ |  | √ |  |  | √ |  | √ | √ | √ | √ | √ | √ |  |  | √ | √ | √ | √ |
| Fibulin 1 [18490682] | √ | √ |  | √ | √ | √ | √ | √ | √ |  |  |  | √ | √ | √ | √ |  | √ | √ |  |  | √ |  | √ |  |  | √ |  | √ | √ | √ | √ | √ | √ |  |  | √ | √ | √ | √ |
| Protein kinase C-binding protein NELL2 [223029476] | √ | √ | √ | √ | √ | √ | √ | √ | √ | √ | √ | √ |  | √ | √ | √ |  | √ | √ |  | √ | √ | √ | √ | √ | √ | √ |  | √ | √ | √ | √ | √ | √ |  |  | √ | √ | √ | √ |
| NOTCH4 protein [187954607] | √ | √ | √ | √ | √ | √ | √ | √ | √ | √ | √ |  | √ |  | √ | √ | √ | √ | √ | √ | √ | √ | √ | √ | √ | √ | √ | √ | √ | √ |  | √ |  | √ |  |  | √ |  |  | √ |
| complement receptor type 2 [54792123] |  | √ |  |  |  |  |  |  |  |  |  |  |  |  |  |  |  |  |  |  |  |  |  |  |  |  |  |  | √ |  |  |  |  |  |  |  |  |  |  | √ |
| dual oxidase 2 precursor variant, partial [62087600] |  |  |  |  |  |  | √ | √ |  |  |  |  |  |  |  |  |  |  |  |  |  |  |  |  |  |  |  |  | √ |  |  |  |  |  |  |  |  |  |  | √ |
| Nidogen-1 / Entactin [115298674] | √ | √ | √ | √ | √ | √ | √ | √ | √ | √ | √ | √ | √ | √ | √ | √ | √ | √ | √ | √ | √ | √ | √ | √ | √ | √ | √ | √ | √ | √ | √ | √ | √ | √ |  | √ | √ | √ | √ | √ |
| CSMD2 protein [62954774] | √ | √ |  | √ | √ | √ | √ | √ | √ | √ |  |  |  | √ | √ | √ | √ | √ | √ | √ | √ | √ | √ | √ | √ | √ | √ | √ |  | √ |  | √ |  | √ |  |  | √ | √ | √ | √ |
| Cysteine-rich with EGF-like Domains 2 (CRELD2) beta [67511376] | √ | √ |  | √ | √ | √ | √ | √ | √ |  |  |  | √ | √ | √ | √ |  | √ | √ |  |  | √ |  | √ | √ |  | √ | √ | √ | √ |  | √ |  | √ | √ |  | √ | √ | √ | √ |
| Endosialin / CD248 [9966885] | √ | √ | √ | √ | √ | √ | √ | √ | √ | √ | √ |  | √ |  | √ | √ | √ | √ | √ | √ | √ | √ | √ | √ |  | √ | √ | √ | √ | √ |  | √ |  | √ |  |  | √ |  |  | √ |
| Epidermal growth factor-like protein 7 [7705889] | √ | √ | √ | √ | √ | √ | √ | √ | √ | √ | √ |  | √ | √ | √ | √ | √ | √ | √ | √ | √ | √ | √ | √ | √ | √ | √ | √ | √ | √ |  | √ |  | √ |  |  | √ | √ | √ | √ |
| Prolow-density lipoprotein receptor-related protein 1 / Alpha-2-macroglobulin receptor / Apolipoprotein E receptor / CD91 [126012562] | √ | √ | √ | √ | √ | √ | √ | √ | √ | √ | √ | √ | √ | √ | √ | √ | √ | √ | √ | √ | √ | √ | √ | √ | √ | √ | √ | √ | √ | √ | √ | √ | √ | √ | √ | √ | √ | √ | √ | √ |
| CUB and sushi domain-containing protein 3 [38045888] | √ | √ |  | √ | √ | √ | √ | √ | √ |  |  | √ |  | √ | √ | √ |  | √ | √ |  |  | √ |  | √ |  |  | √ |  | √ | √ | √ | √ | √ | √ |  |  | √ | √ | √ | √ |
| Thrombospondin-3 [6005902] | √ | √ | √ | √ | √ | √ | √ | √ | √ | √ | √ |  | √ |  | √ | √ | √ | √ | √ | √ | √ | √ | √ | √ | √ | √ | √ | √ | √ | √ |  | √ |  | √ |  |  | √ |  |  | √ |
| Epidermal growth factor-like protein 6 [13124888] |  | √ | √ | √ | √ |  |  |  | √ |  |  |  | √ |  | √ | √ |  |  | √ | √ | √ |  |  |  | √ |  |  |  | √ | √ |  |  |  |  |  |  | √ |  |  | √ |
| Mutant p53 binding protein 1 variant, partial [62087822] | √ | √ |  | √ | √ | √ | √ | √ | √ | √ | √ | √ |  | √ | √ | √ |  | √ | √ | √ | √ | √ | √ | √ | √ | √ | √ | √ | √ | √ | √ | √ | √ | √ | √ |  | √ | √ | √ | √ |
