# Supplementary material for: Amino acid sequence homology between thyroid autoantigens and central nervous system proteins: Implications for the steroid-responsive encephalopathy associated with autoimmune thyroiditis
Source: J Clin Transl Endocrinol. 2021 Nov 6;26:100274. doi: 10.1016/j.jcte.2021.100274 (PMC8609095; doi:10.1016/j.jcte.2021.100274)
Supplement: Supplementary data 4 [file mmc4.doc]

**Supplementary Table 4.** Expression in central nervous system and thyroid, as resulting from a search in the Expression Atlas (https://www.ebi.ac.uk/gxa/home) [31], of the known Hashimoto’s encephalopathy autoantigens. Central nervous system areas whose involvement in Hashimoto’s encephalopathy is reported in literature are highlighted in gray.

|  | Expressed in | | | | | | | | | | | | | | | | | | | | | | | | | | | | | | | | | | | | | | | | | | | | | |
| --- | --- | --- | --- | --- | --- | --- | --- | --- | --- | --- | --- | --- | --- | --- | --- | --- | --- | --- | --- | --- | --- | --- | --- | --- | --- | --- | --- | --- | --- | --- | --- | --- | --- | --- | --- | --- | --- | --- | --- | --- | --- | --- | --- | --- | --- | --- |
| **Protein [Entrez Protein accession number]** | amygdala | basal ganglion | brain | brain meninx | Brodmann (1909) area 24 | Brodmann (1909) area 9 | caudate nucleus | cerebellar hemisphere | cerebellum | cerebral cortex | choroid plexus | diencephalon | dorsal thalamus | dorsolateral prefrontal cortex | dura mater | entorhinal cortex | forebrain | frontal cortex | frontal lobe | globus pallidus | hindbrain | hippocampus | hypothalamus | locus ceruleus | medulla oblongata | midbrain | middle frontal gyrus | middle temporal gyrus | nucleus accumbens | occipital cortex | occipital lobe | parietal lobe | pineal body | pituitary gland | pons | prefrontal cortex | primary visual cortex | putamen | striatum | substantia nigra | telencephalic ventricle | telencephalon | temporal cortex | temporal lobe | thalamus | thyroid |
| Alpha-enolase [P06733] | √ | √ | √ | √ | √ | √ | √ | √ | √ | √ | √ | √ | √ | √ | √ | √ | √ | √ | √ | √ | √ | √ | √ | √ | √ | √ | √ | √ | √ | √ | √ | √ | √ | √ | √ | √ | √ | √ | √ | √ | √ | √ | √ | √ | √ | √ |
| Aldehyde reductase-I [AAF01260] | √ | √ | √ | √ | √ | √ | √ | √ | √ | √ | √ | √ | √ | √ | √ | √ | √ | √ | √ | √ | √ | √ | √ | √ | √ | √ | √ | √ | √ | √ | √ | √ | √ | √ | √ | √ | √ | √ | √ | √ |  | √ | √ | √ | √ | √ |
| Dimethylarginine dimethylaminohydrolase 1 [O94760] | √ | √ | √ | √ | √ | √ | √ | √ | √ | √ | √ | √ | √ | √ | √ | √ | √ | √ | √ | √ | √ | √ | √ | √ | √ | √ | √ | √ | √ | √ | √ | √ | √ | √ | √ | √ | √ | √ | √ | √ |  | √ | √ | √ | √ | √ |
